# A culture method with berbamine, a plant alkaloid, enhances CAR-T cell efficacy through modulating cellular metabolism

**DOI:** 10.1038/s42003-024-06297-0

**Published:** 2024-06-04

**Authors:** Shin-ichiro Takayanagi, Sayaka Chuganji, Masahiro Tanaka, Bo Wang, Saki Hasegawa, Ken Fukumoto, Nariaki Wasano, Makoto Kakitani, Nakaba Ochiai, Yohei Kawai, Tatsuki Ueda, Akihiro Ishikawa, Yuko Kurimoto, Asami Fukui, Sanae Kamibayashi, Eri Imai, Atsushi Kunisato, Hajime Nozawa, Shin Kaneko

**Affiliations:** 1 Kirin Central Research Institute, Kirin Holdings Company, Ltd., 26-1, Muraoka-Higashi 2, Fujisawa, Kanagawa 251-8555 Japan; 2 Shin Kaneko Laboratory, Department of Cell Growth and Differentiation, Center for iPS Cell Research and Application (CiRA), Kyoto University, 53 Kawahara-cho, Shogoin, Sakyo-ku, Kyoto, 606-8507 Japan; 3 Present Address: Biomedical Science Research Laboratories 2, Research Division, Kyowa Kirin Co., Ltd., Tokyo, Japan

**Keywords:** Stem-cell research, Cancer immunotherapy

## Abstract

Memory T cells demonstrate superior in vivo persistence and antitumor efficacy. However, methods for manufacturing less differentiated T cells are not yet well-established. Here, we show that producing chimeric antigen receptor (CAR)-T cells using berbamine (BBM), a natural compound found in the Chinese herbal medicine Berberis amurensis, enhances the antitumor efficacy of CAR-T cells. BBM is identified through cell-based screening of chemical compounds using induced pluripotent stem cell-derived T cells, leading to improved viability with a memory T cell phenotype. Transcriptomics and metabolomics using stem cell memory T cells reveal that BBM broadly enhances lipid metabolism. Furthermore, the addition of BBM downregulates the phosphorylation of p38 mitogen-activated protein kinase and enhanced mitochondrial respiration. CD19-CAR-T cells cultured with BBM also extend the survival of leukaemia mouse models due to their superior in vivo persistence. This technology offers a straightforward approach to enhancing the antitumor efficacy of CAR-T cells.

## Abstract (web summary)

When used in CAR-T cell processing, the plant alkaloid berbamine prolongs the survival of leukemic animals by boosting CAR-T cell viability, memory phenotype, and persistence by modulating cellular metabolism, including mitochondrial respiration.

## Introduction

Recent advancements in adoptive T-cell therapies are revolutionizing cancer treatment options. Chimeric antigen receptor (CAR)-T cells have demonstrated remarkable clinical outcomes against B-cell malignancies, with many studies reporting complete remission (CR) rates of 70−90% for CD19-CAR-T cell therapies in relapsed or refractory B-cell acute leukemia (B-ALL)^1–3^. However, 30−60% of patients who achieved CR experienced disease relapse due to target antigen loss or low CAR-T cell persistence^2^. Additionally, the in vivo persistence and differentiation stages of therapeutic T cells are closely related to the antitumor efficacy and CR rate in clinical trials^4,5^.

Stem cell memory T (T_SCM_) cells may also contribute to this persistence^6,7^. Various approaches, such as controlling cytokine signaling, cellular metabolism, and cellular signaling, including mitogen-activated protein kinase (MAPK) cascades, have been developed to maintain T_SCM_ cells^7^. Combinations of cytokines, such as interleukin (IL)-7, IL-15, and IL-21, are considered critical for controlling the differentiation stages of T cells^8–11^. Terminally differentiated effector T cells primally rely on the glycolysis pathway, while naïve and memory T cells, including T_SCM_ cells, depend on lipid metabolism, including fatty acid oxidization and mitochondria respiration^12,13^. Several types of MAPK signaling are essential for T-cell activation, proliferation, and survival^14^. T cell receptor signaling is regulated by phosphorylation of p38 kinases downstream of ZAP-70^15^ and pharmacological inhibition by the p38 inhibitor BIRB796 has been shown to improve the therapeutic efficacy of antitumor T cell^16^.

Herein, we report a culture method for CAR-T cell processing using berbamine (BBM), a natural compound found in traditional Chinese herbal medicine, aimed at improving in vivo persistence and therapeutic efficacy of CAR-T cells. BBM, a bisbenzylisoquinoline alkaloid derived from *Berberis amurensis*^17^, was identified through chemical screening using induced pluripotent stem cell-derived T (iPS-T) cells. We observed that BBM maintains T cells in a less differentiated state resembling the memory T cell-phonotype. Mechanistic analyses, including demonstrated that BBM enhanced lipid metabolism and downregulated the phosphorylation of p38 in T cells. Thus, these findings have implications for the production of therapeutic T cells for adoptive immunotherapy.

## Results

### Identification of BBM in the chemical screening

**Fig. 1:**
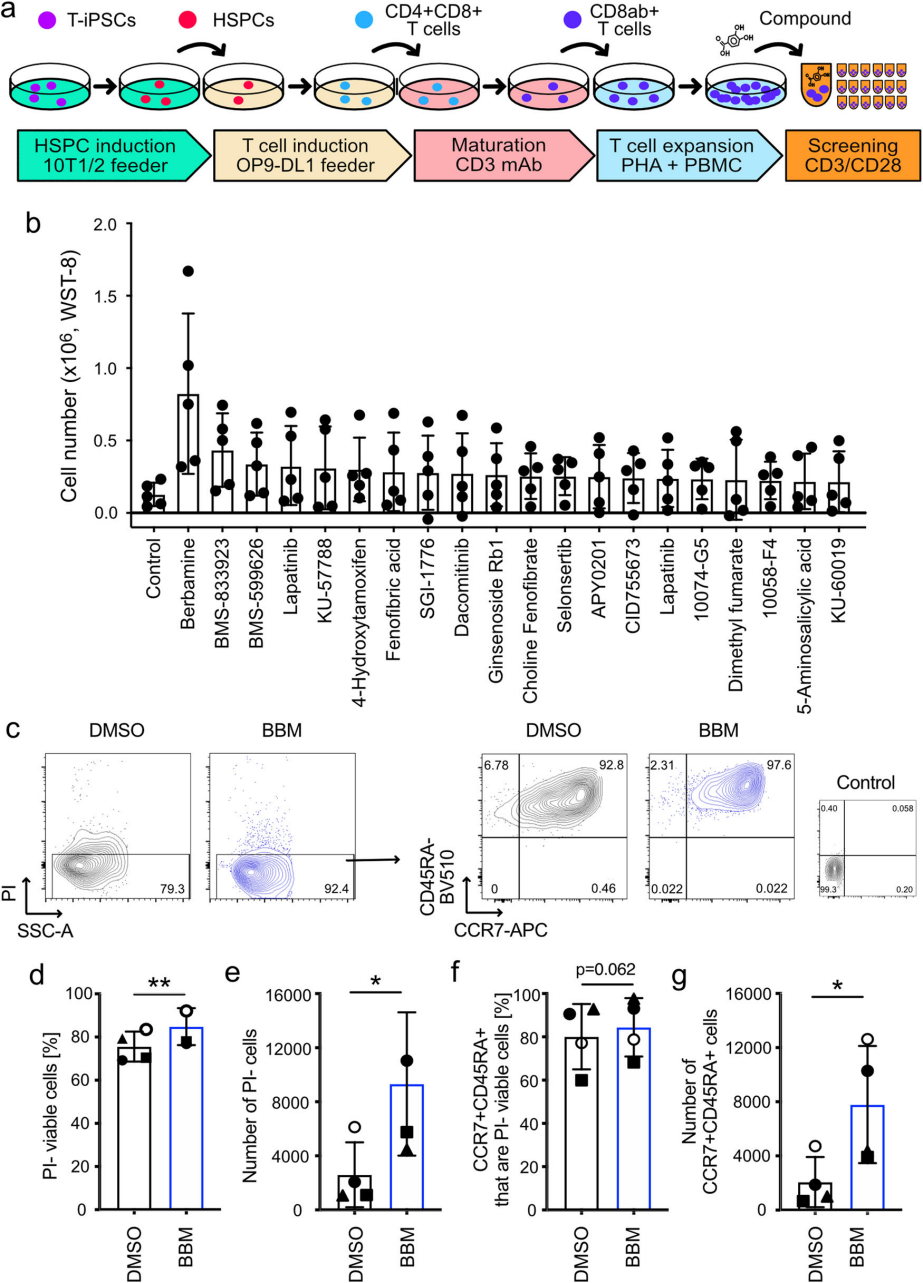
Screening of chemical compounds using the iPS-derived T cells. **a** T cell differentiation scheme from T-iPSCs for screening chemical compounds. Expanded T-iPS-T cells were stimulated with CD3/CD28 microbeads and then incubated with compounds. All data presented in Fig. 1 were acquired 6 d later. **b** Cell numbers based on intracellular dehydrogenase activity. Error bars indicate the standard deviation (SD) of five independent experiments. **c** Representative FACS plots 6 d after the addition of the compounds. **d** Percentage and **e** number of PI-viable cells determined with flow cytometry. **f** Percentage and **g** number of CCR7+CD45RA+ cells. Error bars indicate the SD of four independent experiments (**c**–**f**). Statistical significance is denoted as follows: *p < 0.05; **p < 0.01; and ***p < 0.001 (paired *t*-test).

iPS cells are promising as tools for drug screening as well as a cell source for regenerative medicine. We have previously reported the establishment of T cell-derived induced pluripotent stem (T-iPS) cells and induction systems for generating less differentiated cytotoxic T lymphocytes from T-iPS cells (Fig. 1a)^11,18^. Prior to screening chemical compounds using T-iPS-derived T (T-iPS-T) cells, we monitored the expression of cell surface markers related to T-cell differentiation. We observed partial expression of CCR7, a marker of naive and memory T cells, in T-iPS-T cells (Supplementary Fig. 1a, b, Expansion 1). Over the course of 4 weeks of expansion culture, CCR7 expression gradually decreased (Supplementary Fig. 1c, Expansion 0−2), indicating that the T-iPS-T cells exhibited hierarchical and early memory T cell-like proliferative capacity. We subsequently conducted chemical screening based on the proliferation of T-iPS-T cells (Fig. 1a). Viable cell numbers were determined using a kit that measures intracellular dehydrogenase activities through the reduction of water-soluble tetrazolium salt to formazan. BBM displayed the highest cell number among the 1,080 compounds with validated pharmacological activities that were screened; moreover, the cell number was 6.4-fold higher than that of the control DMSO 6 d after CD3/CD28 stimulation (Fig. 1b). BMS-833923, a Smoothened antagonist, and BMS-599626, a HER1 and HER2 dual inhibitor, also increased the number of cells (Fig. 1b). In this screening, we further characterized the effect of BBM using flow cytometry (Fig. 1c). Cell viability and viable cell number determined with propidium iodide (PI)-negative fraction were increased in BBM-treated T-iPS-T cells (Fig. 1d, e). Expression of CCR7 and CD45RA naïve T cell markers in peripheral blood was maintained in BBM-treated T-iPS-T cells (Fig. 1f)^19^. Numbers of CCR7+CD45RA+ cells were also increased in BBM-treated T-iPS-T cells (Fig. 1g). These data suggest that BBM improves the proliferation of T-iPS-T cells with a less differentiated T-cell phenotype.

### In vitro assessments of BBM-treated primary T cells

**Fig. 2:**
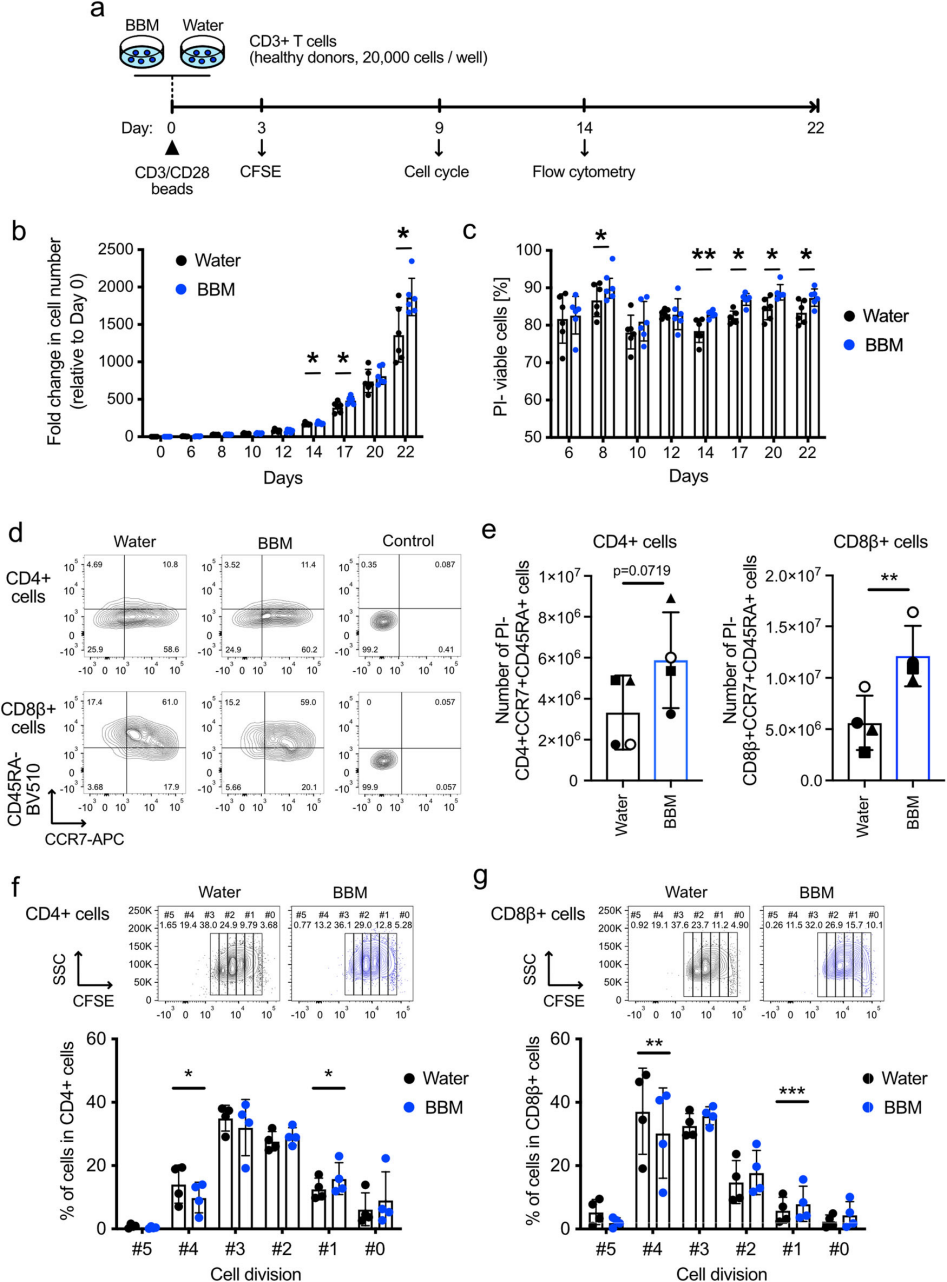
Effects of BBM on human healthy donor-derived T cells. **a** Experimental scheme for characterizing the effect of BBM on primary CD3+ T cells. **b** Fold changes in cell number and **c** viability determined using the standard trypan blue method. Error bars indicate SD from six donors. **d** Representative FACS plots illustrating the expression of CCR7 and CD45RA in CD4+ cells (top) and CD8β+ cells (bottom) on day 14. **e** Estimated numbers of CD4+CCR7+CD45RA+ cells (left) and CD8β+CCR7+CD45RA+ cells (right) in cultured CD3+ T cells calculated from the total cell number and frequencies of the populations that are PI- on day 14. **f**, **g** Results of the CFSE assay in CD4+ (**f**) and CD8β+ (**g**) fractions within CD3+ cells on day 3. Error bars indicate SD from six (**b**, **c**) or four donors (**e**–**g**). Statistical significance is denoted as follows: *p < 0.05; **p < 0.01; and ***p < 0.001 (paired *t*-test).

All the approved CAR-T cell therapies use primary T cells as a cell source^20^. Therefore, we decided to evaluate the effects of BBM on healthy donor-derived primary T cells. The solvent for the BBM solutions was switched from DMSO to water for future clinical use. CD3+ T cells were stimulated with CD3/CD28 microbeads and cultured (Fig. 2a). Cell numbers counted using the trypan blue method were increased in BBM-treated CD3+ T cells on days 14, 17, and 22 after stimulation (Fig. 2b). Viabilities were also increased in BBM-treated CD3+ T cells on days 8 and 14 and at all the subsequent timepoints (Fig. 2c). Flow cytometry on day 14 revealed that the frequencies of CD4+ and CD8β + T cells were comparable between the BBM and water control groups (Supplementary Fig. 2a, b). While the differences in the percentages of CCR7+CD45RA+ cells in CD4+ and CD8β+ cell fractions between BBM- and control water-treated samples were observed (Supplementary Fig. 2c), The BBM treatment resulted in a 2.2-fold increase in the estimated number of CD8β+CD45RA+CCR7+ cells and exhibited a similar trend in the number of CD4+CD45RA+CCR7+ cells, which were affected by the increase in total cell numbers (Fig. 2d, e).

We further monitored the cell division using CFSE, cell cycle phase, and the expression of T cell exhaustion markers. Primary CD3+ cells were stimulated as described above and co-stained with CD4 and CD8β. As a result of flow cytometry of CFSE-labeled CD3+ cells on day 3 after stimulation, percentages at cell division #1 were increased in BBM-treated CD4+ and CD8β+ fractions (Fig. 2f, g). In contrast a decrease at cell division #4 was observed in BBM-treated cells, suggesting the possibility of slower cell division in BBM-treated T cells (Fig. 2f, g). BBM treatment did not affect the cell cycle phase in CD4+ and CD8β+ fractions at 14 d after stimulation (Supplementary Fig. 3a, b) nor the expression levels of PD-1, TIM-3, and LAG-3, T cell exhaustion markers, at 9 d after stimulation (Supplementary Fig. 4a, b). Collectively, these in vitro characterizations using human primary T cells revealed that BBM increases the proportion of phenotypically less differentiated T cells with superior viability.

### Improvement of the in vivo persistence by BBM

**Fig. 3:**
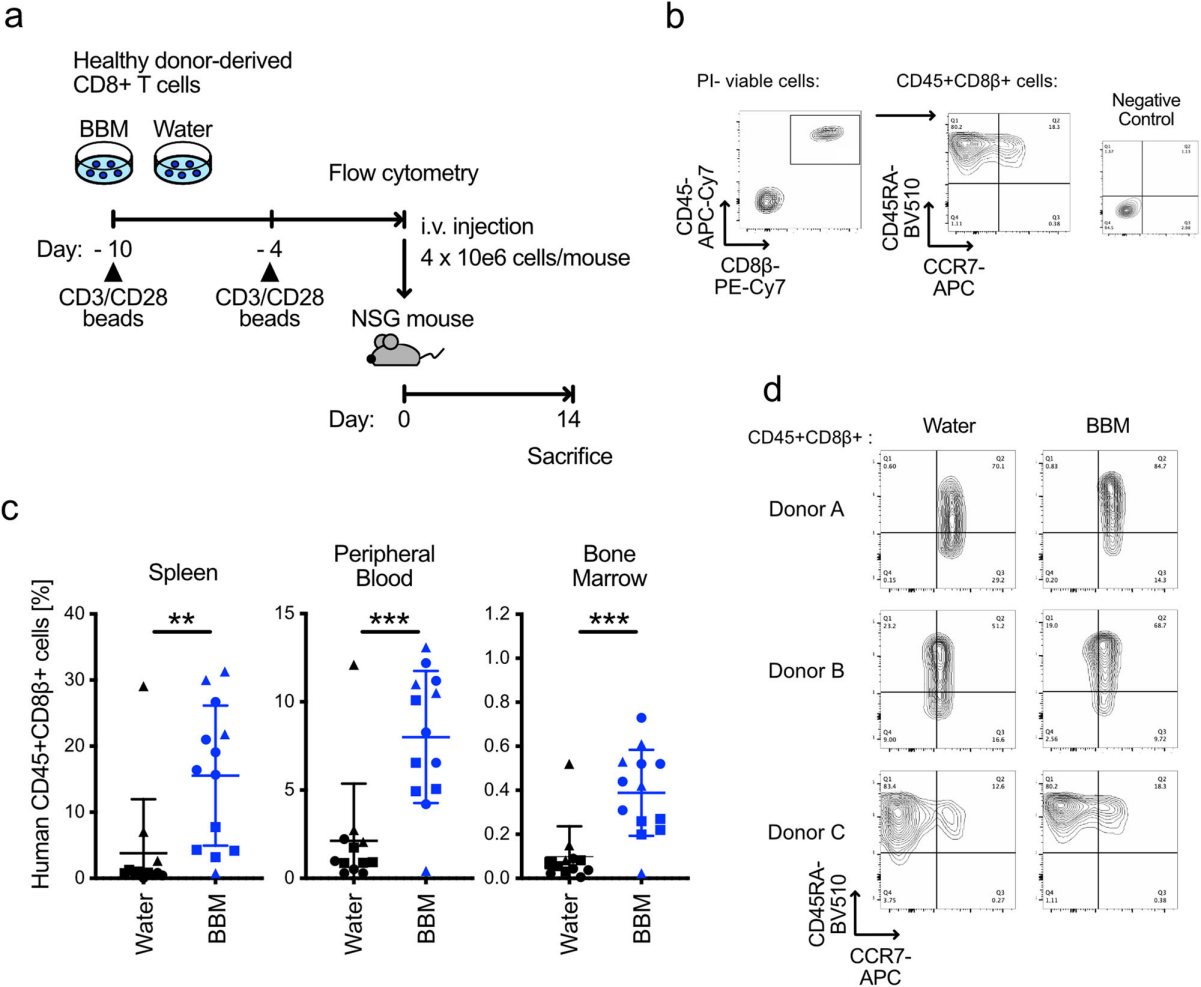
In vivo persistence of primary T cells derived from healthy donors. **a** Experimental scheme designed to assess the in vivo persistence of BBM-treated primary CD8+ T cells. **b** Gating strategy employed to determine the expression of CCR7 and CD45RA in the CD45+CD8β+ fractions. **c** Frequencies of injected CD45+CD8β+ cells in the spleen (left), peripheral blood (center), and bone marrow (right) on day 14. Each data point represents one injected mouse and its corresponding donors: circle; donor A, triangle; donor B, square; donor C. All error bars indicate SD. Statistical significance is denoted as follows: *p < 0.05; **p < 0.01; and ***p < 0.001 (unpaired *t*-test). **d** Representative flow cytometry plots of CCR7 and CD45RA in CD45+CD8β+ cells in the spleen samples.

The correlation between in vivo persistence and T-cell differentiation is well-established^4–7^. Based on the in vitro characteristics of BBM-treated primary T cells, such as improved viability and increased numbers of CCR7+CD45RA+ less differentiated cells, we hypothesized that BBM treatment improves the in vivo persistence of T cells. To test this hypothesis, we cultured primary CD8+ T cells in the presence of BBM for 10 d and injected them into NSG mice as xenoreactive T cells are present in primary T cells at a certain rate (Fig. 3a)^21^. Cultured T cells from all three donors formed a single CCR7+CD45RA+ population, and no significant difference in the percentage CCR7+CD45RA+ cells [%] was not observed at 10 d after stimulation (Supplementary Fig. 5a, b). Fourteen days after injection, the frequencies of the injected CD45+CD8β+ T cells were higher in the spleen, peripheral blood, and bone marrow in the BBM treatment group compared to the control group (3.6-fold, 2.9-fold, and 2.8-fold, respectively) (Fig. 3b, c). T cells engrafted in the spleen commonly maintained the expression of CCR7 and CD45RA in both BBM- and control-treatment groups, with partial downregulation of CCR7 observed in one donor (Fig. 3d). These result suggest that while BBM can affect T cells during culture, BBM-treated T cells behave normally once engrafted in the mouse body, with increased frequencies of engraftable T cells in the BBM-treated group.

Prolonged culture revealed that BBM improved viability at 2 weeks after stimulation and BBM-treated CD8+ T cells derived from all four donors were expandable for at least 5 weeks. In contrast, control T cells derived from two of the four donors stopped proliferating after 3 or 4 weeks after the stimulation (Supplementary Fig. 5c, d). These improved viabilities by BBM in culture might contribute to the increased cell numbers and higher in vivo persistence, which are key factors for adoptive T cell immunotherapy.

### Gene expression analysis in stem cell memory T cells

As demonstrated above, BBM treatment led to the maintenance of a less differentiated phenotype and conferred improved viability and proliferative capacity both in vitro and in vivo, reminiscent of the self-renewal capacity that is a definitive feature of stem cells. In the field of adoptive T-cell immunotherapy, T_SCM_ cells, a memory T cell subset with self-renewal capacity, can improve clinical outcomes^6^. Therefore, we evaluated the effect of BBM on T_SCM_ cells.

**Fig. 4:**
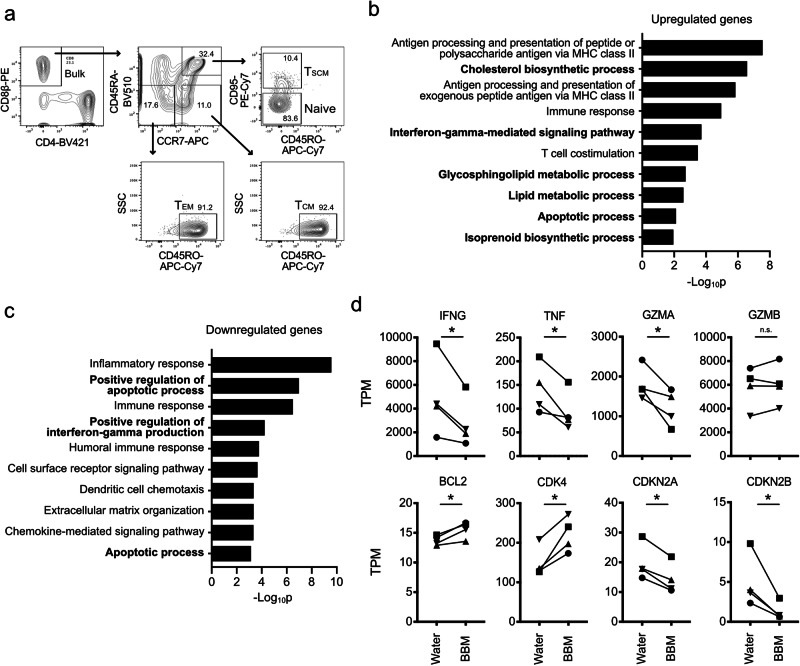
Gene expression analysis in BBM-treated stem cell memory T cells. **a** Gating strategy for FACS to purify the naïve and memory T cell fractions from healthy donor-derived CD8+ cells. **b**, **c** Gene ontology analysis of DEGs in BBM-treated T_SCM_ cells. Upregulated (**b**) or downregulated (**c**) genes from RNA-sequencing results on day 12. Genes were queried in the DAVID functional annotation database. **d** Transcripts per million (TPM) values of cell cycle-related and effector function-related genes. Statistical significance is denoted as follows: *p < 0.05; **p < 0.01; and ***p < 0.001 (*n* = 4, paired *t*-test). T_SCM_ stem cell memory T, T_CM_ central memory T, T_EM_ effector memory T.

As the first step to elucidate the mechanism of BBM, we purified CD8β+ T cells into CD45RA+CCR7+CD45RO-CD95- naïve T cells, CD45RA+CCR7+CD45RO-CD95+ T_SCM_ cells, CD45RA-CCR7+CD45RO+ T_CM_ cells, and CD45RA-CCR7-CD45RO+ effector memory T (T_EM_) cells and cultured them with BBM (Fig. 4a). Among the four fractions, the naïve and T_SCM_ fractions as well as bulk CD8β+ cells showed some increases in cell numbers within 9−14 d of the culture, whereas the T_CM_ and T_EM_ fractions did not (Supplementary Fig. 6). These data indicate that the high proliferative capacities of T_SCM_ and naïve T cells were reproduced in our culture conditions^6^, and BBM increased their cell numbers when highly purified with FACS.

To gain mechanistic insights into BBM, RNA-sequencing was performed on T_SCM_ cells, and 431 genes were extracted as differentially expressed genes (DEGs). The gene ontology of upregulated and downregulated DEGs was analyzed using the DAVID functional annotation database^22^. Among the upregulated genes, the cholesterol biosynthetic process displayed the second lowest *p* value, and the glycosphingolipid metabolic process, lipid metabolic process, and isoprenoid biosynthetic process were also ranked in the top ten GO biological processes (Fig. 4b). In contrast, metabolism-related categories were ranked among the downregulated genes (Fig. 4c).

GO analysis also suggested that the genes related to the “Interferon-gamma-mediated signaling pathway” and “Apoptotic process” were included in both up- and down-regulated genes. In fact, the expression of *IFNγ* was downregulated in BBM-treated T_SCM_ cells, as were other effector function-related genes such as *TNFA* and *GZMA*, consistent with their upregulation during the maturation from T_SCM_ cells to T_EM_ cells^6^. Regarding the apoptotic process, the expression level of *Bcl-2*, an anti-apoptotic molecule associated with CD8+ T cell survival and highly expressed in CD8+ memory T cells, was upregulated in BBM-treated T_SCM_ cells (Fig. 4d)^23,24^. Moreover, upregulation of a cyclin-dependent kinase *CDK4* and downregulation of cell cycle inhibitor genes, *CDKN2A* (p16) and *CDKN2B* (p15), were observed^25^. These data suggest that BBM modulates various biological processes.

### BBM modulates metabolic status and MAPK signaling in stem cell memory T cells

**Fig. 5:**
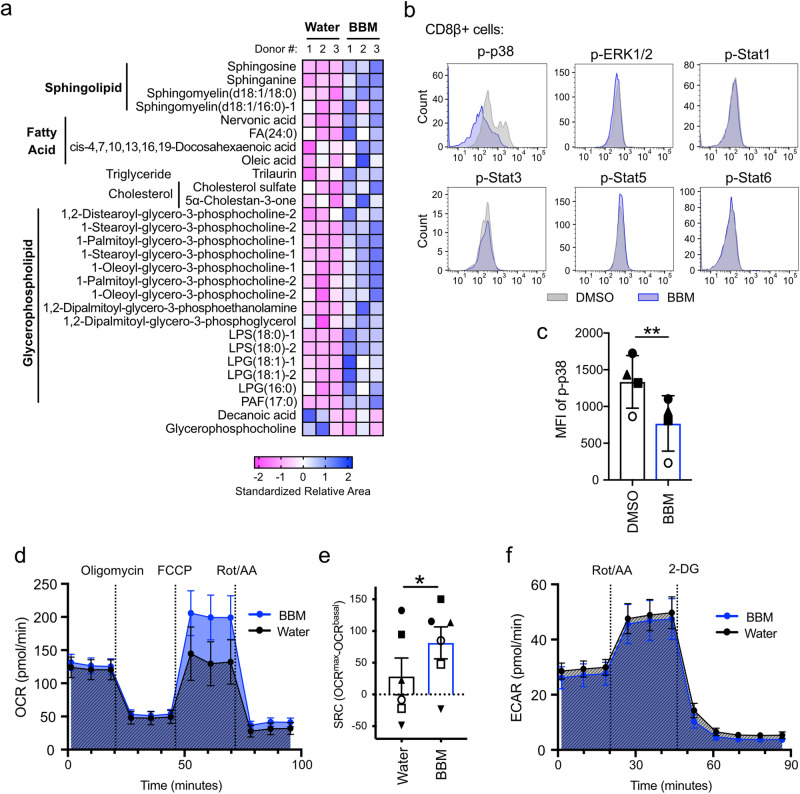
Effect of BBM on metabolism and signaling pathways in primary T cells. **a** Metabolomics of BBM-treated T_SCM_ cells from healthy donor-derived CD8+ cells. Lipids commonly upregulated or downregulated in all three donors are shown. **b** Representative flow cytometry plots of Phosflow assay in BBM-treated CD3+ cells on day 10. Phosphorylated proteins in CD8+ T cells are shown. **c** Mean fluorescence intensity (MFI) of phosphorylated p38 in CD8+ T cells. Error bars indicate SD from four donors. **d** Oxygen consumption rate (OCR) across time for BBM-treated CD3+ T cells. **e** Spare respiratory capacity (SRC) calculated by OCRmax-OCRbasal. **f** Extracellular acidification rates (ECAR) across time for BBM-treated CD3+ T cells. Error bars indicate the standard error of the mean from six donors (**d**–**f**). Statistical significance is denoted as follows: *p < 0.05; **p < 0.01; and ***p < 0.001 (paired *t*-test) (**c**, **e**).

Because the RNA-sequencing results indicated that BBM treatment affected the various cellular metabolic processes, we performed a metabolomics analysis of T_SCM_ cells using both capillary electrophoresis (CE) and liquid chromatography–mass spectrometry (LC-MS) to monitor global changes by BBM at the metabolite level. As expected from the GO analysis, cholesterol sulfate, a derivative of cholesterol, was commonly increased in all three donors. Sphingolipids (sphingosine, sphinganine, sphingomyelin [d18:1/18:0], etc.), fatty acids (nervonic acid, FA[24:0], oleic acid etc.), a triglyceride (trilaurin), cholesterols (cholesterol sulfate and 5α-cholestan-3-one), and several glycerophospholipids were also increased in BBM (Fig. 5a), suggesting that BBM broadly enhances lipid metabolism. Levels of metabolites related to nucleic acids and amino acids were also changed in BBM-treated T_SCM_ cells (Supplementary Fig. 7a). Primary CD3+ T cells that were cultured in combinations of BBM and inhibitors of lipid metabolic pathways—fumonisin B1, a competitive inhibitor of ceramide synthase; cerulenin, an inhibitor of fatty acid synthase; and methyl-beta-cyclodextrin, an inhibitor of fatty acid and cholesterol synthesis—did not increase CD8β+CCR7+CD45RA+ cell numbers (Supplementary Fig. 7b), supporting the activation of these metabolic pathways by BBM.

To explore the signaling pathway that upregulates lipid metabolism in BBM-treated T cells, we monitored the phosphorylation of STAT proteins and MAPK signaling proteins because these pathways are known to regulate the metabolism under the TCR signaling. Interestingly, the phosphorylation of p38 MAPK was downregulated by BBM (Fig. 5b, c), but other proteins tested (ERK1/2 and STAT1/3/5/6) remained unchanged (Supplementary Fig. 7c). Recently, two groups reported that MAPK inhibition in human CD8+ T cells improved the antitumor efficacy of adoptive immunotherapy by modulating metabolic states^16,26^. To gain deeper insights into the links between MAPK pathways and BBM, we analyzed mitochondrial respiration because p38 inhibition and MEK inhibition commonly upregulate mitochondrial respiration capacities^16,26^. As a result, BBM-treated primary CD3+ T cells demonstrated higher maximal oxygen consumption rates (OCR) after the addition of FCCP, an uncoupler of oxidative phosphorylation in mitochondria, consistent with the characteristics of memory T cells (Fig. 5d and Supplementary Fig. 7d)^24^. The spare respiratory capacity (SRC), an indicator of the adaptive capacity to stress conditions, was higher in BBM-treated T cells (Fig. 5e)^27^. Hence, glycolytic rates remained unchanged in BBM-treated CD3+ T cells (Fig. 5f and Supplementary Fig. 7e). Overall, the modulation of cellular metabolism via the inhibition of the MAPK pathway could be a possible mechanism of action for BBM.

### Effector functions of BBM-treated CAR-T cells

**Fig. 6:**
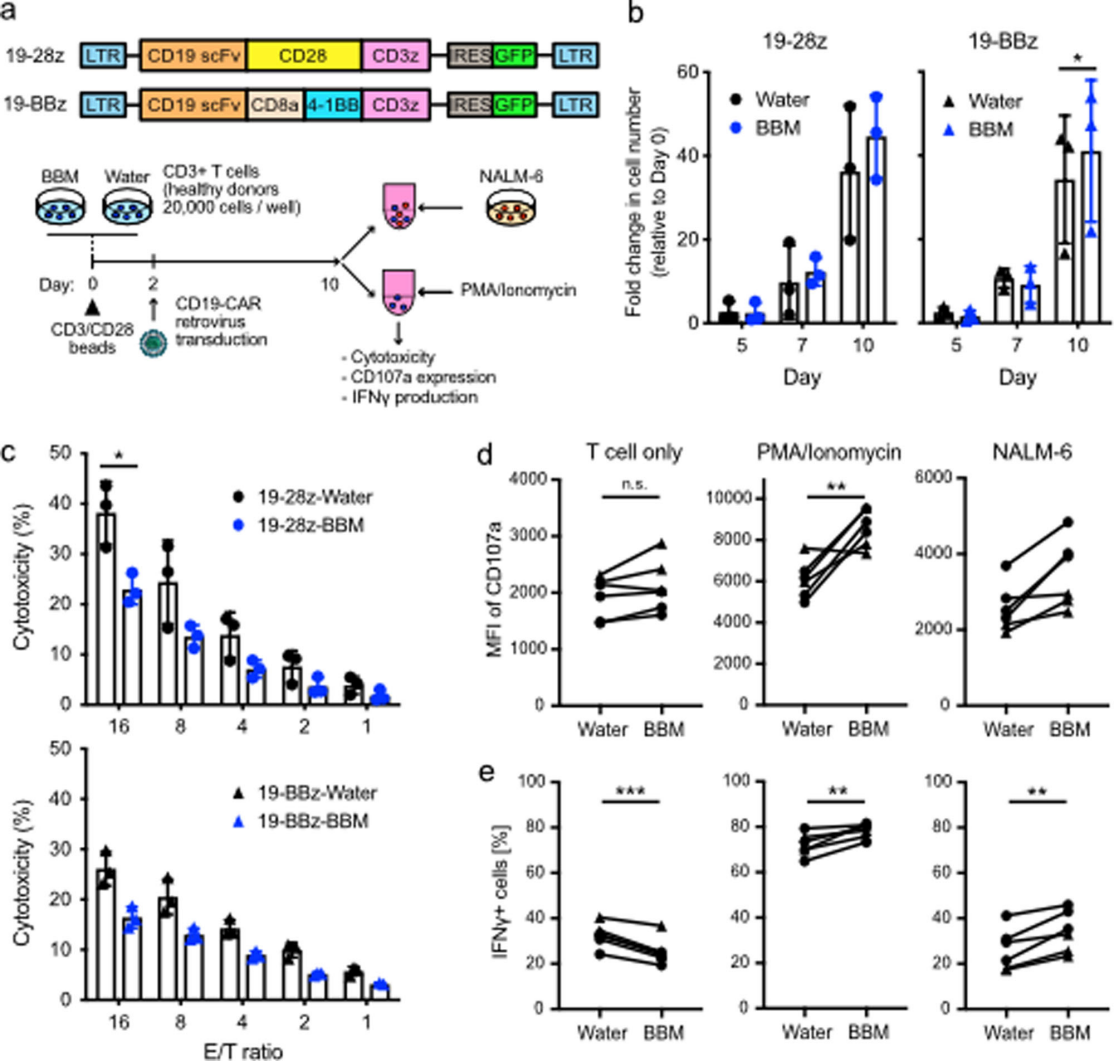
Effector functions of second-generation CAR-T cells. **a** CD19-CAR retrovirus vectors (top) and an experimental scheme for in vitro assays. **b** Assessment of cell proliferation among CD3+ T cells transduced with CD19-CAR (CAR-T cells). Fold expansion rates compared to Day 0 were shown. **c** Evaluation of in vitro cytotoxicity of CAR-T cells against CD19-expressing NALM6 cells. E/T ratio effector/target ratio. Error bars indicate SD from three donors (**b**, **c**). **d** Expression of CD107a and **e** IFNγ production in CAR-T cells that were stimulated with PMA plus Ionomycin (PMA/Ionomycin), NALM6 cells, or unstimulated control (T cell only). Circle; 19-28z, triangle; 19-BBz. Statistical significance is denoted as follows: *p < 0.05; **p < 0.01; and ***p < 0.001 (paired *t*-test).

As mentioned in the Introduction section, successful treatments with CAR-T cell therapy have transformed the treatment of B-cell malignancies. To evaluate the effect of BBM on CAR-T cells, we compared second-generation CD19-CAR constructs with a CD28 costimulatory domain (19-28z) or a 4-1BB costimulatory domain (19-BBz) (Fig. 6a). While the proliferation of BBM-treated CAR-T cells did not differ from that of control CAR-T cells (Fig. 6b), BBM-treated CAR-T cell viability was higher than that of control CAR-T cells at day 10 (Supplementary Fig. 8a). The percentage of CAR-T cells expressing CD4 and CD8β as well as CCR7 and CD45RA memory T cell markers were similar between BBM-treated CAR-T cells and control CAR-T cells (Supplementary Fig. 8b, c). Additionally, the expression of T-cell exhaustion markers (PD-1, LAG-3, and TIM-3) was comparable between BBM-treated CAR-T cells and control CAR-T cells (Supplementary Fig. 8d).

Next, we compared in vitro cytotoxicity against CD19-expressing NALM6 cells. BBM treatment attenuated the specific lysis of both 19-28z (control: 38.3%, BBM: 23.0%, effector/target (E/T) ratio = 16) and 19-BBz (control: 26.1%, BBM: 16.4%, E/T ratio = 16) compared to their respective control CAR-T cells (Fig. 6c). According to the hierarchical model of human T cell differentiation, more differentiated cells exhibit higher cytotoxicity^28^. Thus, BBM-treated CAR-T cells were less differentiated.

Because the in vitro assay encompasses the cumulative cytotoxic activity mechanisms, we decided to scrutinize these mechanisms in greater depth. CAR-T cells were incubated with NALM6 cells to monitor the response to CAR-mediated stimulation and with PMA plus Ionomycin to evaluate the potential for cytokine production (Fig. 6a). The MFI of CD107a, also known as lysosomal-associated membrane protein-1 (LAMP-1), an indicator of degranulation function in cytotoxic lymphocytes, was higher in BBM-treated CAR-T cells when incubated with PMA/Ionomycin and with NALM6 cells, but not when CAR-T cells were incubated alone (Fig. 6d). IFNγ production was also higher in BBM-treated CAR-T cells when incubated with NALM6 cells, but not when CAR-T cells were incubated with PMA/Ionomycin or under CAR-T cell alone conditions (Fig. 6e). These data indicate that the presence of BBM during the culture of CAR-T cells enhances the potential of effector functions. Notably, increased IFNγ production after antigen-specific or CAR-mediated stimulation was reported when T cells were cultured in the presence of MAPKi or MEKi, that increased the less differentiated memory T cells^16,26^. Collectively, these data indicate that CAR-T cells exhibit less differentiated characteristics when cultured in the presence of BBM.

### BBM-treated CAR-T cells enhance the survival of leukemia mouse models through superior persistence

Less differentiated T cells potentially demonstrate superior in vivo persistence and therapeutic effect compared to the differentiated effector T cells^6,28^. Based on the in vitro data indicating that the BBM-treated CAR-T cells were less differentiated (Fig. 6) and the higher engraftment of primary T cells in NSG mice (Fig. 3), we evaluated whether in vivo antitumor efficacy could be improved by using BBM. To establish the appropriate experimental settings in the standard luciferase-expressing NALM6/NSG mouse models^29^, we injected various cell numbers of CAR-T cells, which were confirmed to express GFP and CD19-CAR protein, 4 days after the NALM6 injection (day 0) (Supplementary Fig. 9a, b). The antitumor effect of both control CAR-T cells and BBM-treated CAR-T cells was observed in the mice transplanted with 1 × 10^5^ cells/mouse and 2 × 10^5^ cells/mouse on day 13 (Supplementary Fig. 9c).

**Fig. 7:**
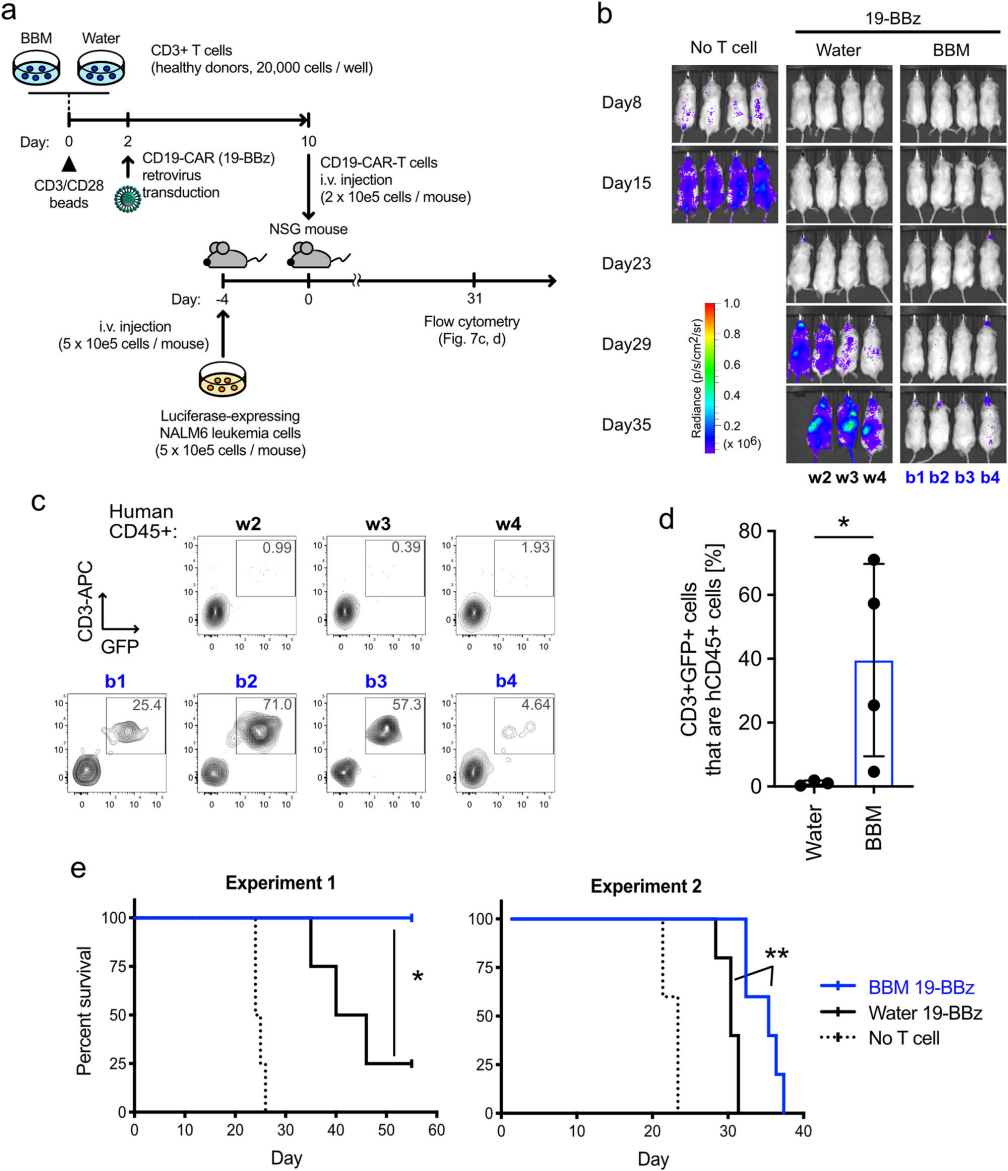
Berbamine improves the therapeutic effect of CAR-T cells. **a** Experimental scheme of the in vivo assays. Bioluminescence in Luciferase-expressing NALM6 cells were monitored to measure the amount of leukemia burden. CD19-CAR with 4-1BB costimulatory domain (19-BBz) was transduced into primary CD3+ T cells and cultured with BBM or water. After 10 days of cultivation, CD19-CAR-T cells (2 × 10^5^ cells/mouse) were injected into each leukemia mouse model. **b** IVIS images to monitor NALM6 cells. The scale bar represents the bioluminescence signal in radiance (p/s/cm^2^/sr). **c** Representative flow cytometry plots and **d** percentages CD3+ GFP+ cells that are hCD45+ cells in peripheral blood on day 31. Statistics: Mann–Whitney test. **e** Kaplan–Meier survival analysis. Statistics: log-rank Mantel–Cox test. Statistical significance is denoted as follows: *p < 0.05; **p < 0.01; and ***p < 0.001.

Then we evaluated the therapeutic effects, including survival rates, for longer periods at 2 × 10^5^ cells/mouse, based on the test above (Fig. 7a). In Experiment 1, in vivo imaging demonstrated that both water- and BBM-treated CAR-T cells effectively controlled the growth of NALM6 cells until day 23 compared to the no T cell control group (Fig. 7b). NALM6 cells rapidly grew in mice injected with water-treated CAR-T cells on day 29 (Fig. 7b). Regarding the survival rate, mice injected with PBS (No T cell control) died around days 24–26, consistent with a previous report^29^. To compare the in vivo persistence of the CAR-T cells, peripheral blood samples collected from the viable mice were analyzed with flow cytometry on day 31. Human CD3+ T cells with GFP, a transduction marker for CD19-CAR, were detected only in mice injected with BBM-treated CAR-T cells, suggesting improved persistence with culture in the presence of BBM (Fig. 7c, d). As a result of subsequent monitoring, three out of four mice injected with water-treated CAR-T cells died by day 42. In contrast, mice injected with BBM-treated CAR-T cells survived until at least 51 days after the CAR-T cell injection (Fig. 7e). In Experiment 2, the effects of BBM were evaluated using T cells derived from another healthy donor. Mice in the no T cell control group died around day 20–22, 4 days earlier than in Experiment 1, suggesting that tumor growth in Experiment 2 was more aggressive. While mice injected with water-treated CAR-T cells died by day 30, mice injected with BBM-treated CAR-T cells demonstrated a longer survival rate, dying from day 31–36 (Fig. 7e). Overall, treating CAR-T cells with BBM improved the therapeutic effects of CAR-T cell therapy.

## Discussion

In this study, we established a novel T cell culture method utilizing BBM, which not only increased cell numbers and enhanced cell viability, but also promoted the in vivo persistence of primary T cells, including CD19-CAR-T cells. The presence of BBM facilitated the maintaince of memory T cell properties, such as surface marker expression and mitochondrial respiration, consequently improving the in vivo antitumor effect. Our findings prompt further discussions on screening methodologies, elucidating the mechanism of action of BBM, and exploring future perspectives.

While the correlation between in vivo persistence and antitumor efficacy is well accepted, the underlying mechanisms remain elusive. Inhibitors targeting signaling molecules like Wnt/β-catenin^6,30^ and PI3K/AKT/mTOR^31^ have shown promise in regulating T_SCM_ cell activities and enhancing in vivo persistence. In another study, functional screening of chemical probes targeting defined epigenetic factors revealed that bromodomain and extra-terminal motif (BET) inhibition in augmenting antitumor efficacy through the maintenance of T_SCM_ cells^21^. In contrast to these targeted approaches, we performed a cell-based screening from unbiased chemical libraries consisting of 1080 pharmacology-validated compounds to uncover novel mechanisms regulating T_SCM_ cells.

One notable feature of our screening method is the use of T-iPS-T cells with a memory phenotype. Each T cell clone in the human body has different TCRs and histories of antigen recognition, and this heterogeneity might reduce the reliability of a cell-based screening when small numbers of primary T cells were used for the screening. Therefore, we used iPS cells as the cell source to generate homogeneous T cells. To apply the iPS cell screening results to primary T cells, it is crucial that the T cell induction of iPS cells mimics the natural developmental process. For this reason, we avoided using any genetic approaches, such as introducing TCRs or transcription factors related to the T cell lineage commitment^32–34^. Furthermore, because the iPS cells in this study were established from T cells, the expression levels and copy numbers of TCRs were physiological. Finally, recognizing the importance of cytokine combinations such as IL-7, IL-15, IL-21, and JAK-STAT signaling^10,35,36^, we employed five cytokines (IL-7, IL-12, IL-15, IL-18, and IL-21) to maintain T cells in the less differentiated state^11^.

Among the basic characteristics of BBM, the most observed effect of BBM in cell types such as T-iPS-T cells, primary T cells, and CAR-T cells was the improvement of cell viability. Given the homogeneous cell population with CCR7 and CD45RA expression in cultured T cells with BBM, it is considered that BBM enhances overall cell proliferation by improving cell viability, leading to an increase in T cell numbers with a memory phenotype. The downregulation of effector-associated genes and reduced cytotoxicity in BBM-treated T cells align with the theory of hierarchical differentiation of memory T cells^6,28^. Therefore, BBM-treated T cells exhibit a less differentiated state.

Metabolic changes observed in RNA-seq and metabolomics, such as the idea that T cell effector function demands glycolysis^12,13,37^, and that T cell memory functions are regulated by mitochondrial respiration and fatty acid oxidation^24,38^, are also important consensuses in the field of adoptive immunity. Hence, the balance between glycolysis and other metabolic pathways has been considered when controlling the differentiation status of T cell subsets^39^.

Additionally, the downregulation of p38 MAPK phosphorylation by BBM was consistent with a previous report that identified p38 inhibition based on T cell expansion and T_SCM_ cell phenotype screening to improve adoptive T cell therapy^16^. Pharmacological inhibition of MEK1/2, a central player in MAPK cascades, was reported to reprogram CD8+ T cells to T_SCM_ cells and to delay cell division. Enhanced mitochondrial respiration determined by OCR was observed in BBM-treated T cells, as commonly reported in p38 and MEK inhibition papers and CD8+ T cell memory formation^32^. Additionally, enhanced IFNγ production and superior in vivo antitumor effects were observed between BBM and MEK inhibition or p38 inhibition. We concluded that MAPK cascades are modulated by BBM. One speculative molecular mechanism of BBM is the direct binding to the ATP-binding domain of calmodulin-kinase II gamma (CamKIIγ) protein^40^. CamKII activates p38 in macrophages, and its inhibition attenuates MAPK, including p38 phosphorylation and mTOR signaling, in a cadmium-induced neuronal cell death model^41,42^. This hypothesis in T cells would be worth evaluating in future studies.

Clinical outcomes of adoptive T cell immunotherapies are influenced by the manufacturing conditions as well as CAR constructs^43^. Our study indicates that BBM improves the survival rate of approved CAR-T cell therapy. Because BBM was simply added to the media, this effect can be easily tested and could potentially revive CAR-T cells that were unsuccessful in clinical development due to low efficacy without requiring additional genetic engineering. It is worth noting that numerous groups have proposed new CAR constructs and culture conditions, and there is significant demand for CAR-T cells to treat cancers other than CD19-expressing B-cell malignancies^44^. However, the availability of established in vivo mouse models for various cancer types is quite limited. To evaluate long-term antitumor efficacy in vitro, a repetitive stimulation experiment provides an alternative system. Therefore, we aim to establish such a system and compare the effect of BBM in these technologies as part of our future studies.

Superior viability in BBM-treated T cells would confer longer-term proliferative capacities during manufacturing. While many groups have tried to maintain the quality and quantity of T_SCM_ cells during CAR-T cell manufacturing, short-term culture is one of the trends in this field. For instance, the manufacturing process time of the T-Charge^TM^ platform was less than 2 days and would be a great platform for patients in need of quick autologous CAR-T therapy^45^. Our protocol for CAR-T cell culture with BBM was 10 days in this study, and exploring the optimization of a shorter culture period is worthwhile, similar to the T-Charge^TM^ platform for autologous settings, aiming to shorten the vein-to-vein time. On the other hand, there is a demand for longer culture times in allogeneic CAR-T cell therapy approaches, such as iPS cells, aiming to manufacture large numbers of pre-prepared vials for ready-to-infuse at low cost. Because iPS cells can be differentiated into T cells in a wide variety of methods, we believe that encouraging users to test this technology on their own will help provide many patients with treatment opportunities.

## Methods

### iPS cells

T-iPS cells (Clone TkT3v1-7^18^, from Univ. of Tokyo) were maintained on irradiated mouse embryonic fibroblasts (MEFs) using Dulbecco’s Modified Eagle’s Medium/Nutrient Mixture F-12 Ham (DMEM/F-12; Sigma, D6421) supplemented with 20% Knockout Serum Replacement (KSR; Life Technologies, 10828-028), 1% l-Glutamine-Penicillin-Streptomycin solution (PSG; Sigma, G1146), 1% MEM Non-Essential Amino Acids Solution (NEAA; Life Technologies, 11140-050), 2-Mercaptoethanol (0.1 mM, Gibco, 21985-023), and Fibroblast Growth Factor basic (5 ng/mL, Sigma, 068-04544).

### T-cell induction from T-iPS cells and culture

[HSPC induction] Hematopoietic stem/progenitor cells (HSPCs) were induced as previously described with slight modification^18^. Clumps of iPS cells were transferred onto 10T1/2 feeder cells (RIKEN BRC) to form sacs containing HSPCs and cultured in Iscove’s Modified Dulbecco’s Medium (IMDM; Sigma, I3390) supplemented with 15% fetal bovine serum (FBS; CORNING, 35-076-CV), 1% PSG, 1x Insulansferrin-Selenium (ITS-G; Gibco, 41400-045), Monothioglycerol (MTG; 0.45 mM, Nacalai Tesque, 33709-62), l-Ascorbic Acid 2 Phosphate Sesquimagnesium Salt Hydrate (PAA; 50 µg/mL, Nacalai Tesque, 03420-52) and Recombinant Human VEGF 165 Protein (VEGF; 20 ng/mL, R&D Systems, 293-VE/CF) [Sac medium] (Day 0). The cells were incubated in 5% O_2_, 5% CO_2_. On days 7, 10, and 12, the medium was changed to Sac medium supplemented with Recombinant Human SCF Protein (SCF; 30 ng/mL, R&D Systems, 255-SC) and Recombinant Human Flt3-Ligand (Flt3L; 10 ng/mL, Peprotech, 300-19), and the cells were incubated in 20% O_2_, 5% CO_2_.

[T cell induction] On day 14, HSPCs were collected and co-cultured on OP9-DL1 (RIKEN BRC) feeder cells in OP9 medium (see “Cell lines” section below) containing Recombinant Human IL-7 (IL-7; 1 ng/mL, Peprotech, 200-07), Flt3L (10 ng/mL), PAA (50 µg/mL), and ITS-G^18^. The cells were reseeded onto fresh OP9-DL1 cells on day 17 and every 6 days thereafter. The medium was changed every 3 days.

[Maturation] Mature CD8α+CD8β+ cells were induced from CD4+CD8+ cells as previously described^11^. On day 38, the cells were stimulated with anti-CD3 antibody (OKT3, 1 µg/mL, Thermo Fisher Scientific, 16-0037-38) in OP9 medium supplemented with IL-7 (10 ng/mL), Flt3L (10 ng/mL), PAA (50 µg/mL), and ITS-G for 3 days. The generated T cells were harvested and cultured on RetroNectin (2 µg/mL, Takara Bio, T100B) in OP9 medium supplemented with IL-7 (10 ng/mL), Flt3L (10 ng/mL), Recombinant Human IL-21 (IL-21; 10 ng/mL, Peprotech, AF-200-21), PAA (50 μg/mL), and ITS-G for 6 days. Mature CD8β+CD5+CD1α-CD336- cells were sorted using a FACSAria-II cell sorter (BD Biosciences) as CD8αβ+ T cells [T-iPS-T cells].

[T cell expansion] In order to obtain enough numbers of T-iPS-T cells (usually initial cell numbers are on the order of 10^3^−10^4^ cells in our system), the cells were expanded to reach 10^5^-fold as described below. T-iPS-T cells were co-cultured with irradiated PBMC (40 Gy) in OP9 medium supplemented with IL-7 (5 ng/mL), IL-15 (5 ng/mL), Pan Caspase Inhibitor Z-VAD-FMK (10 μM, R&D Systems, AF-200-21), PAA (50 μg/mL), ITS-G and Phytohemagglutinin (PHA; 2 µg/mL, Wako, 161-15251) for 14 days. The medium was replenished every 2–3 days.

[T-iPS-T culture] Expanded T-iPS-T cells were stimulated with Dynabeads Human T-Activator CD3/CD28 (CD3/CD28 microbeads; beads/cells = 3, Thermo Fisher Scientific, 1132D) in OP9 medium supplemented with IL-7 (5 ng/mL), IL-12 (50 ng/mL, Millipore, GF420), IL-15 (5 ng/mL), IL-18 (50 ng/mL, MBL, B001-5), IL-21 (20 ng/mL), Z-VAD-FMK (10 μM), PAA (50 µg/mL), and ITS-G [Assay Media]. The medium was replenished every 2–3 days, and cell passaging was performed every 2–6 days. The cells were restimulated and counted every 14 days for long-term culture.

### Cell lines

10T1/2 cells were cultured on a 0.1% gelatin-coated dish in Basal Medium Eagle (Life Technologies, 21010-046) supplemented with 10% FBS and 1% PSG. OP9-DL1 cells were cultured in Minimum Essential Medium α (α-MEM; Life Technologies, 11900-024) supplemented with 15% FBS and 1% PSG [OP9 medium]. NALM6 cells (ATCC) and NALM6-Luciferase (Luc)-Kusabiraorange (KO) (see “Viral production” section) were cultured in RPMI-1640 (Sigma, R8758) supplemented with 10% FBS and 1% PSG. GP2-293 cells　(Takara Bio) and FLYRD18 cells (ECACC) were maintained in Dulbecco′s Modified Eagle′s Medium (DMEM; Life Technologies, 11965-092) supplemented with 10% FBS and 1% PSG. Mycoplasma contamination was confirmed to be negative before each animal experiment or on a monthly basis.

### Compounds and screening

Chemical libraries were purchased from MedChemExpress (Apoptosis Compound Library: HY-L003; Cell Cycle/DNA Damage Compound Library: HY-L004; JAK/STAT Compound Library: HY-L008; MAPK Compound Library: HY-L010; NF-kappaB Compound Library: HY‐L014; PI3K/Akt/mTOR Compound Library: HY‐L015; and Wnt/Hedgehog/Notch Compound Library: HY-L020).

Expanded T-iPS-T cells were stimulated with CD3/CD28 microbeads and seeded into a 96-well plate (1500 cells/well) in RPMI-1640 medium supplemented with 15% FBS, 1% PSG, IL-7, IL-12, IL-15, IL-18, IL-21, Z-VAD-FMK, PAA, and ITS-G as described in [T-iPS-T culture]. Compounds were added at 1 µM, and DMSO (SIGMA, D2650-100 mL) was added as a control. Six days after stimulation, cell proliferation was measured using the Cell Count Reagent SF (Nacalai Tesque, 07553-15) and SoftMaxPro 5.X (Molecular Devices, 450 nm/650 nm). Standard curves were generated using frozen T-iPS-T cells. Berbamine dihydrochloride (1 µM, Sigma, 547190-1 G), Methyl-beta-cyclodextrin (10 µM, FUJIFUILM WAKO, 320-84252), Cerulenin (1 µM, WAKO, 17397-89-6), and Fumonisin B1 (20 nM, WAKO, 116355-83-0) were used for further assays.

### Cell sorting from primary T cells

Human CD3+ and CD8+ T cells were purified from cryopreserved PBMCs (Cellular Technologies Ltd., CTL-UP1) derived from healthy donors by negative magnetic selection using a Pan T Cell Isolation Kit (Miltenyi Biotec, 130-096-535) or CD8+ T Cell Isolation Kit (Miltenyi Biotec, 130-096-495). Naïve and memory T cell fractions were sorted as shown in Fig. 4a. Briefly, CD8+ T cells purified from frozen human PBMCs were stained with anti-CD8β-PE (Beckman Coulter, IM2217U), anti-CD4-BV421 (BioLegend, 317434), anti-CD45RA-BV510 (BioLegend, 304142), anti-CD45RO-APC-Cy7 (BioLegend, 304228), anti-CCR7-APC (BioLegend, 353214), and anti-CD95-PE-Cy7 (BioLegend, 305622) antibodies. Naïve T cells (CD8β+CCR7+CD45RA+CD45RO-CD95-), T_SCM_ cells (CD8β+CCR7+CD45RA+CD45RO-CD95+), T_CM_ cells (CD8β+CCR7;CD45RA-CD45RO+) and T_EM_ cells (CD8β+CCR7-CD45RA-CD45RO+) were sorted on a BDFACSAriaII cell sorter^19^.

### Primary T cell culture

T cells were stimulated using CD3/CD28 microbeads in Assay Media (beads/cells = 3). The cells were cultured in the presence of BBM (1 µM) or control (DMSO or water). The culture media were replenished every 2–3 days with the Assay Media containing BBM (1 µM) or control (DMSO or water). For the in vivo persistence assays (Fig. 3), CD8+ T cells were restimulated 6 days after the first stimulation (beads/cells = 1). Cells were counted using a TC20 cell counter (Bio-Rad).

### Flow cytometry

Cells were harvested in appropriate tubes or 96-well plates. After spindown, supernatants were removed and cells were resuspended in staining medium (PBS containing 2% FBS). After spindown, antibodies diluted in staining medium (total volume: 50 uL/sample; anti-CD8β antibodies, 2 uL/sample; other antibodies, 1 uL/sample) were added and stored in a refrigerator for 30 min. Cells were washed with staining medium and then resuspended in staining medium containing PI (for tube, 200 to 300 uL; for 96-well plate, 150 uL/well; Invitrogen, P3566). Cells were analyzed on FACSAria or FACS LSR (BDBioscience). The following antibody panels were used:

- Immunophenotyping of T-iPS-T cells A (Supplementary Fig. 1b): anti-CD8β-PE, anti-CD45RA-BV510, CD45RO-APC-Cy7, anti-CCR7-APC, anti-CD95-PE-Cy7, and anti-CD62L-FITC (BioLegend, 304804)

—Immunophenotyping of T-iPS-T cells B (Supplementary Fig. 1b): anti-CD8β-PE, anti-CD27-APC (BioLegend, 302810), and anti-CD28-Brilliant Violet 421 (BioLegend, 302930)—T-iPS-T cells on Day 6 (Fig. 1c-g) and cultured primary CD8+ cells on Day 0 (Supplementary Fig. 5a, b): anti-CD8β-PE, anti-CD45RA-BV510, and anti-CCR7-APC

- Frequencies of CD4+ and CD8+ cells in primary T cells and CAR-T cells (Supplementary Figs. 2a, b,  8b): anti-CD8β-PE-Cy7 (Invitrogen, 25-5273-42), anti-CD4-BV421, and anti-CD3-APC (BioLegend, 353214).

- Memory T cell marker expression in primary T cells (Fig. 2d, e and Supplementary Fig. 2c): anti-CD8β-PE, anti-CD4-FITC (BioLegend, 317408), anti-CD45RA-BV510, and anti-CCR7-APC.

- T cell exhaustion markers (Supplementary Fig. 4): anti-CD8β-PE, anti-CD4-FITC, anti-PD-1-BV421 (BioLegend, 329920), anti-TIM-3-PE-Cy7 (BioLegend, 345014), and anti-LAG-3-APC-eFluor™ 780 (Thermo Fisher Scientific, 47-2239-42).

- Fresh samples from xenograft mouse (Fig. 3c): anti-CD45-APC-Cy7 (BioLegend, 304014), and anti-CD8β-PE-Cy7.

- Frozen splenocytes from xenograft mouse (Fig. 3b, d): anti-CD8β-PE-Cy7, anti-CCR7-APC, anti-CD45-APC-Cy7, anti-CD4-BV421, and anti-CD45RA-BV510.—Memory T cell marker expression in primary CAR-T cells (Fig. 2d, e and Supplementary Fig. 2c): anti-CD8β-PE, anti-CD4-BV421, anti-CD45RA-APC-H7 (BDBioscience, 561212), and anti-CCR7-APC.

- T cell exhaustion markers in primary CAR-T cells (Supplementary Fig. 8d): anti-CD8β-PE, anti-CD4-APC (BioLegend, 317416), anti-PD-1-BV421, anti-TIM-3-PE-Cy7, and anti-LAG-3-APC-eFluor™ 780.

- CAR-T cell detection in mouse leukemia model: anti-CD45-BV510 (Biolegend, 304036), and anti-CD3-APC.

### CFSE assay

CD3+ cells stained with carboxyfluorescein succinimidyl ester (CFSE; 1 µM, Invitrogen, C34554) were stimulated with CD3/CD28 microbeads as above (beads/cells = 3) and cultured for 3 d. After staining with anti-CD3-APC (1.5 uL/sample), anti-CD4-BV421 (1 uL/sample), and anti-CD8β-PE (2 uL/sample) antibodies, the cells were analyzed using a BD LSRFortessa X-20 flow cytometer and FlowJo 10.7.1 software.

### Cell cycle

Cultured CD3+ T cells (as described in “Primary T cell culture”) were stained with anti-CD3-APC (3 uL/sample), anti-CD4-FITC (2 uL/sample), and anti-CD8β-PE (4 uL/sample) antibodies and sorted using a SONY MA900 cell sorter at 9 d after stimulation. Purified CD4+ and CD8β + T cells were stained with Cell Cycle Assay Solution Blue (Dojindo Molecular Technologies) following the manufacturer’s instructions. DNA content was measured using the SONY MA900 cell sorter. Data were analyzed with the FlowJo 9.9.6 software based on the Dean-Jett-Fox model.

### RNA-sequencing

Total RNA was purified using the RNeasy Mini kit (QIAGEN, 74104). Libraries were prepared from total RNA (RIN >9.6, Agilent 2200 TapeStation) using a SMART-seq v4 Ultra Low Input RNA kit (Takara Bio, Z4888N), Nextera XT DNA Library Preparation Kit (Illumina, FC-131-1024) and Nextera XT Index Kit v2 (Illumina) and quantified using a Fragment Analyzer (Agilent). More than 55 million reads were sequenced on a NovaSeq6000 using NovaSeq Control Software v1.4.0, Real-Time Analysis v3.3.3. and bcl2fastqv2 v2.20. Expected counts and transcript per million (TPM) were calculated from Fastq files on RSEM v1.3.0 (and STAR v2.6.0a). Expected counts and transcript per million (TPM) are provided in Supplementary Data 2 and 3, respectively. The human genome GRCh37 and corresponding annotation Ensembl were obtained from Illumina’s iGenomes project. DEGs were extracted from protein-coding genes using expected counts on edgeR_3.30.3 R version 4.0.2 and uploaded onto DAVID v6.8 for gene ontology term enrichment analysis^46^.

### Metabolomics

Samples were prepared according to manuals from Human Metabolome Technologies, Inc. and analyzed using CE-MS and LC-MS methods.

[CE-MS] Cells (1.24–2.07 million) were washed with 10 mL of 5% (w/w) mannitol solution and resuspended in 800 µL of methanol. After 30 seconds of vortex treatment, 0.55 mL of fresh Internal Standard Solution (10 µM, provided by HMT) was added to the samples. After centrifugation (2300×*g*, 4 °C, 5 min), the supernatants were loaded onto ultrafiltration columns, washed with water, and centrifuged (9100×*g*, 4 °C, 5 h). [LC-MS] Cells (1.24–2.07 million) were washed with 10 mL of 5% (w/w) mannitol solution and resuspended in 1.0 mL of ethanol containing 5 µM of fresh Internal Standard Solution. The samples were kept at −80 °C until shipment.

### Phosflow

Cultured CD3 + T cells (as described in “Primary T cell culture”) were stained with anti-CD8-AF488 (BDBioscience, 557696) and AF647-labeled anti-phosphoprotein antibodies in the BD Phosflow T Cell Activation Kit (Human) (BDBioscience, 560750) at 10 days after stimulation. Samples were analyzed using the BD LSRFortessa X-20 flow cytometer. Expression levels of phosphorylated proteins in CD8+ cells were analyzed.

### OCR measurement

OCR was measured on an XFe 24 extracellular flux analyzer (Agilent Technologies Inc., Santa Clara, CA, USA) using a Seahorse XF Cell Mito Stress Test Kit (Agilent, 103015-100). Briefly, CD3+ T cells cultured for fourteen days were seeded on the collagen-coated XFe 24 cell cultured plate (2 × 10^5^ cells/well, 100 uL/well, five technical replicates) and incubated at 37 °C for 20 min. After the addition of the assay media (400 uL/well), cells were incubated at 37 °C for 20 min. Baseline OCR was measured, and then 0.5 μM oligomycin, 0.5 μM fluoro-carbonyl cyanide phenylhydrazone (FCCP), and 0.5 μM rotenone/antimycin A were sequentially added into each well. The acquired data were analyzed using Wave Desktop 2.4 (Agilent Technologies).

### ECAR measurement

ECAR was measured on an XFe 24 extracellular flux analyzer using Seahorse XF Glycolytic Rate Assay Kit (Agilent, 103344-100). Briefly, CD3+ T cells cultured for 14 days were seeded on the collagen-coated XFe 24 cell cultured plate (2 × 10^5^ cells/well, 100 uL/well, five technical replicates) and incubated at 37 °C for 5 min. After the addition of the assay media (400 uL/well), cells were incubated at 37 °C for 45 min. Baseline ECAR was measured, and then 5 μM rotenone/antimycin A and 50 mM 2-deoxy-glucose (2-DG) were sequentially added into each well. The acquired data were analyzed using Wave Desktop 2.4.

### Vector construction and viral production

GFP in CMV-Luciferase-EF1a-copGFP-T2A-Puro Lentivector (System Biosciences, BLIV513PA-1) was replaced with Kusabiraorange (KO) amplified from the phKO1-S1 vector (MBL, AM-V0044M). Obtained CMV-Luciferase-EF1a-hKO1-T2A-Puro lentiviral plasmid DNA and ViraPower Packaging Mix DNA (Invitrogen, K497500) were transfected into 293FT cells, and the concentrated supernatant was infected into NALM6 cells (NALM6-Luc-KO cells). The chimeric antigen receptor gene, CTL019, was synthesized from patent US20140271635 and designated as 19-BBz (Supplementary Table 1). The transmembrane domain and the costimulatory domain (4-1BB) in 19-BBz were replaced with CD28, as previously described and designated as 19-28z (Supplementary Table 1)^35^. Synthesized 19-BBz and 19-28z cDNAs were inserted into pMYs-ires-gfp retroviral vector (pMYs-IG; Cell Biolabs, RTV-021). pMYs-IG vectors and VSV-G expression plasmid DNA (Takara Bio, 631530) were transfected into GP2-293 cells, and the obtained supernatants were transferred onto FLYRD18 retroviral packaging cells. Retroviruses were concentrated from the FLYRD18-derived supernatant using a Lenti-X Concentrator (Clontech, 631231), resuspended in α-MEM media and kept in a −80 °C deep freezer until use.

### Retroviral transduction

Retrovirus-encoding CAR genes were transduced into CD3+ T cells derived from human peripheral blood. A 96-well plate was coated with Retronectin for 2 h at room temperature or overnight at 4 °C. CD3+ T cells 2 days after CD3/CD28 stimulation were transduced with the concentrated retroviral supernatants by centrifugation on the RetroNectin-coated plate. Cells were cultured for 8 days from the transduction (total 10 days). Transduction was confirmed using monitoring of GFP and staining with biotinylated-protein-L (GenScript, M00097) and subsequent streptavidin-APC (Biolegend, 405207) on the FACS LSR.

### CD107a and cytokine detection

T cells were stimulated with 50 ng/ml PMA (Wako, 162-23591) plus 1 μg/ml Ionomycin (Ion; Wako, 095-05831) or co-cultured with NALM6 cells at a 1:1 ratio in a 96-well plate for 5 h in 100 uL of RPMI-1640 supplemented with glutamine, 1× Monensin (BioLegend, 420701), 10% FBS, and anti-CD107a-APC antibody (BioLegend, 328620, 1 ul). Then, the cell surface was stained with anti-CD19-PE-Cy7 (BioLegend, 302216) and anti-CD3-BV510 (BioLegend, 300448). Samples were washed and fixed with Fixation Buffer (BioLegend, 420801) for 20 min. Samples were washed twice with Permeabilization Wash Buffer (BioLegend, 421002) and were stained with anti-IFNγ-APC-Cy7 (BioLegend, 506524) for 20 min. The washed and resuspended cells were analyzed using BD FACSAria-II. Expression levels of CD107a and IFNγ in CD19-CD3+ cells were analyzed.

### In vitro cytotoxicity assay

Cytotoxicity was examined using the N-SPC Non-Radioactive Cellular Cytotoxicity Assay Kit (Technosuzuta, N-SPC-01). Briefly, target NALM6-Luc-KO cells were labeled with BM-HT Reagent for 15 min, mixed with effector CD19-CAR-T cells at various effector/target ratios, and incubated in RPMI-1640 media supplemented with 10% FBS and 1% PSG for 40 min. After spindown, 25 µL of the supernatant samples containing leaked HT chelate were mixed with 250 µL Eu Solution to form an HT/Eu complex. The HT/Eu complex (200 µL) was transferred to a fresh 96-well plate. Time-resolved fluorescence was measured using a microplate reader Nivo (PerkinElmer, excitation; 320 nm, emission; 615 nm).

### Mice

NOD.CG-Prkdc scid Il2rg tm1Wjl /SzJ (NSG) mice (Charles River Laboratories Japan) were housed under controlled humidity and a light/dark cycle in a specific-pathogen-free facility.

For the in vivo persistence assays, CD8+ T cells (~4 × 10^6^ cells, see above) were intravenously (i.v.) injected into male NSG mice (6–11 weeks old). Two weeks after the injection, the mice were sacrificed, and samples (spleen, peripheral blood and bone marrow) were analyzed.

### Mouse leukemia model

A total of 5 × 10^5^ NALM6-Luc-KO cells were i.v. injected into female NSG mice (6–8-week-old) to establish a mouse leukemia model (day −4). Body weight was measured before the tumor injection (100%). Four days later, CAR-T cells (0.2, 1, or 2 × 10^5^ cells/mouse) were i.v. injected (day 0). Body weight was monitored every day, and 20% body weight loss was applied as the humane endpoint for sacrifice. Peripheral blood cells were harvested to detect CAR-T cells and analyzed as described above (see “Flow Cytometry”). Tumor burden was monitored by in vivo bioluminescence imaging using IVIS SPECTRUM and Living Image 4.7.3 software (PerkinElmer).

### Statistics and reproducibility

All data are presented as means ± SD unless otherwise specified in the figure legend. All statistics were performed using a two-tailed Student’s *t*-test, otherwise described in the figure legend. Values of *p* < 0.05 were considered significant.

### Study approval

The entire study was conducted in accordance with the Declaration of Helsinki and approved by the Kyoto University School of Medicine Ethical Committee (No. G590). All ethical regulations relevant to human research participants were followed. Informed consent was obtained. All animal experiments were approved by and performed in accordance with the Animal Review Board at Kyoto University. We have complied with all relevant ethical regulations for animal use.

### Reporting summary

Further information on research design is available in the Nature Portfolio Reporting Summary linked to this article.

### Supplementary information

Supplementary Information

Description of Additional Supplementary Files

Supplementary Data 1

Supplementary Data 2

Supplementary Data 3

Reporting summary

## Data Availability

The source data underlying the graphs in the paper can be found in Supplementary Data 1. Supplementary Data 2 contains the analysed data from the RNA-seq experiment (Expected counts, Fig. 4b, c). Supplementary Data 3 shows the analysed data from RNA-seq (TPM, Fig. 4d). All RNA-seq data have been deposited in the GEO under the accession codes GSE 264206. The other datasets generated and analysed during this study are available from the corresponding author upon reasonable request.
