# Supplementary material for: A culture method with berbamine, a plant alkaloid, enhances CAR-T cell efficacy through modulating cellular metabolism
Source: Commun Biol. 2024 Jun 4;7:685. doi: 10.1038/s42003-024-06297-0 (PMC11150386; doi:10.1038/s42003-024-06297-0)
Supplement: Supplementary file 1 — Supplementary Information [file 42003_2024_6297_MOESM1_ESM.pdf]

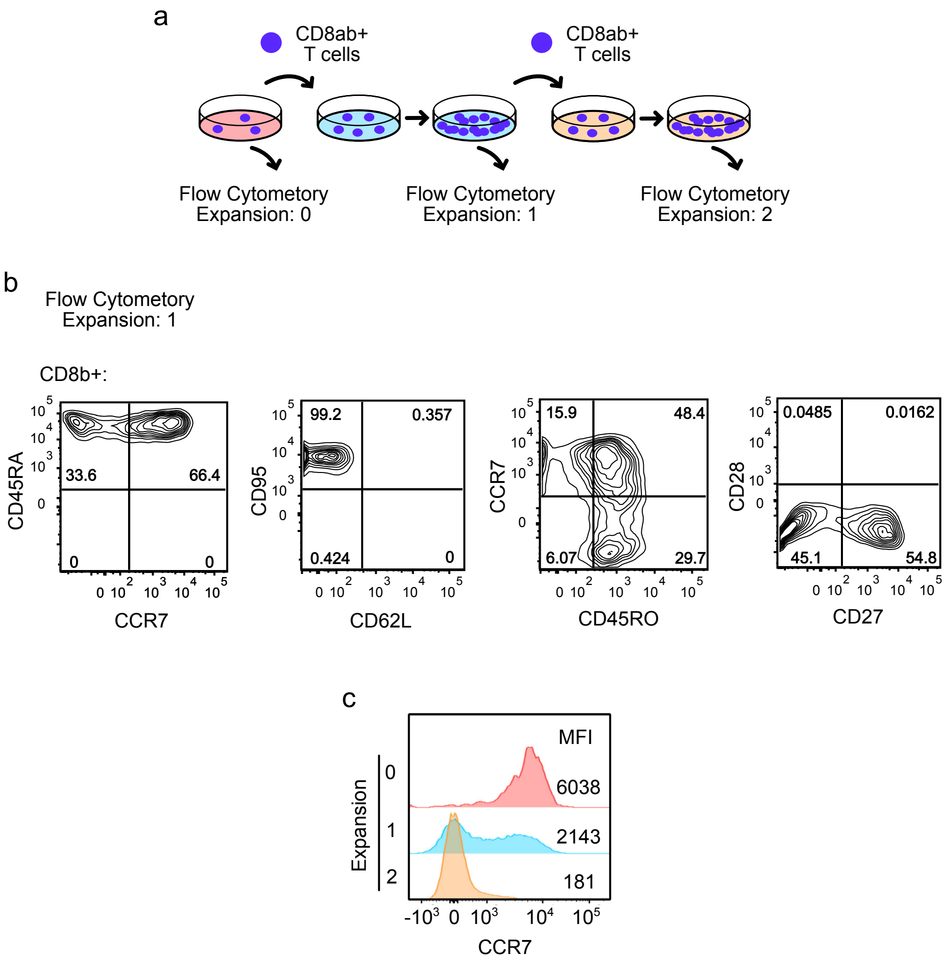

Supplementary Figure 1

(a) Experimental scheme to compare the expression of T cell markers.

(b) Representative flow cytometry plots of T-iPS-T cells at Expansion 1.

(c) Expression levels of CCR7 at Expansions 0, 1, and 2.

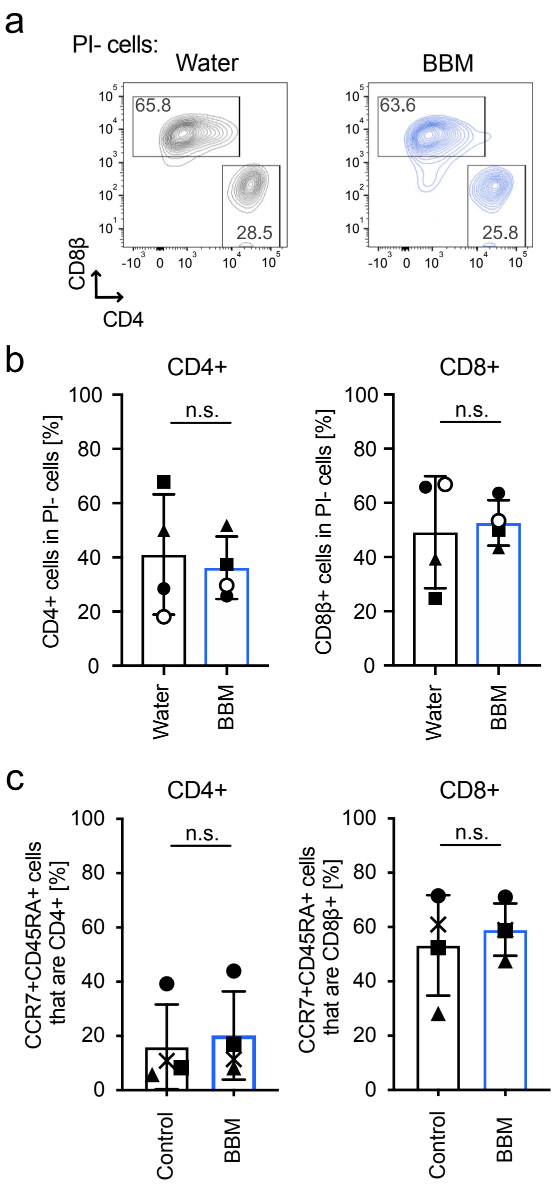

Supplementary Figure 2
(a) Representative flow cytometry plots that depict expression of CD4 and CD8β in cultured CD3+ cells.
(b) Frequencies of CD4+ (left) and CD8β+ (right) cells.
(c) Frequencies of CCR7+CD45RA+ cells in CD4+ (left) and CD8β+ (right) cell populations.
Each data point in graphs represents one healthy donor. Error bars indicate SD from four donors. Statistical significance is denoted as follows: *, $p < 0.05$; **, $p < 0.01$; ***, $p < 0.001$; and 'n.s.', not significant (paired t-test).

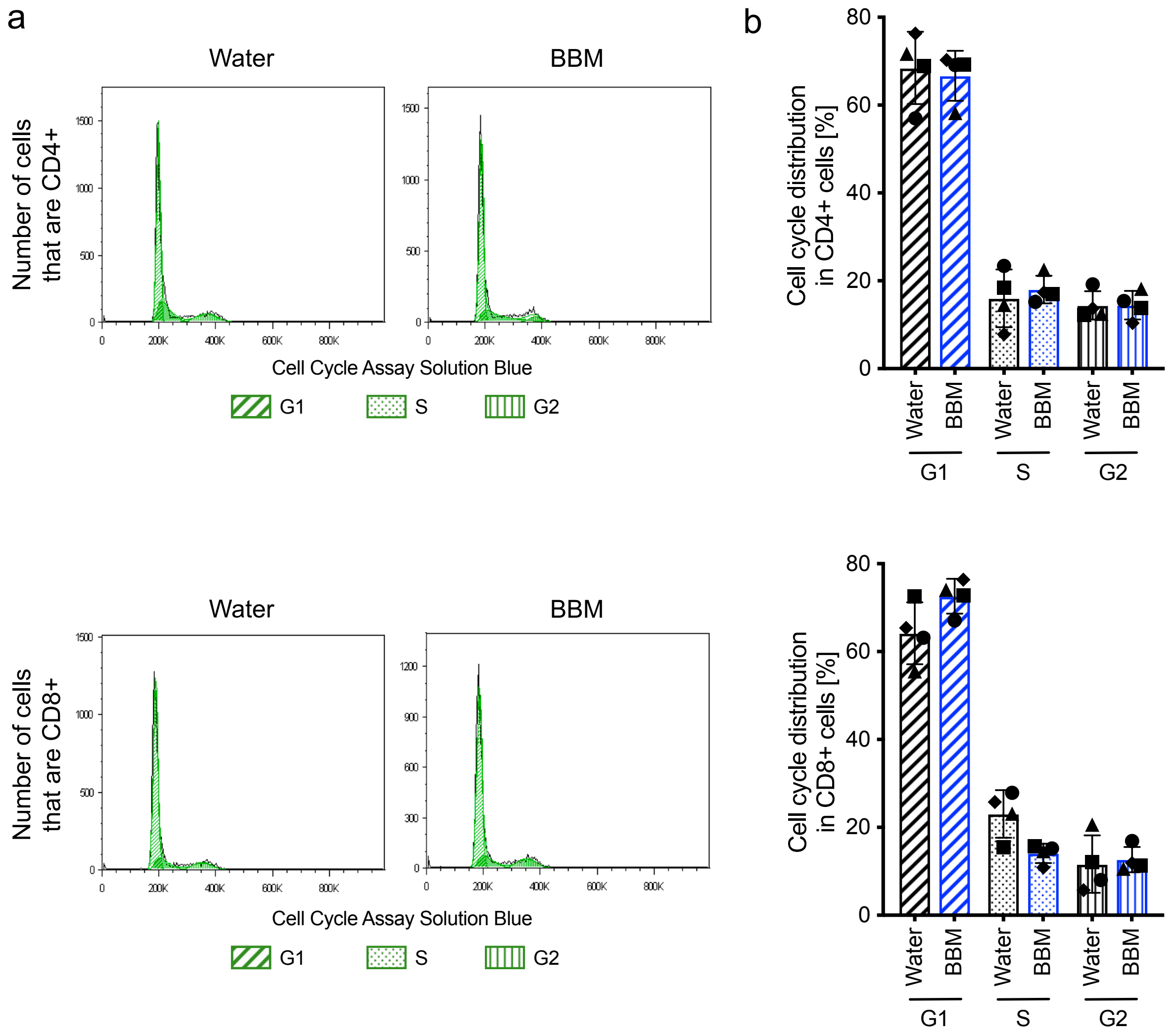

Supplementary Figure 3
(a) Representative FACS plots to determine cell cycle phases in CD4+ (top) and CD8β+ (bottom) cell populations.
(b) Distribution of cell cycle phases in CD4+ (left) and CD8β+ (right) cell populations. Error bars indicate SD from four donors.

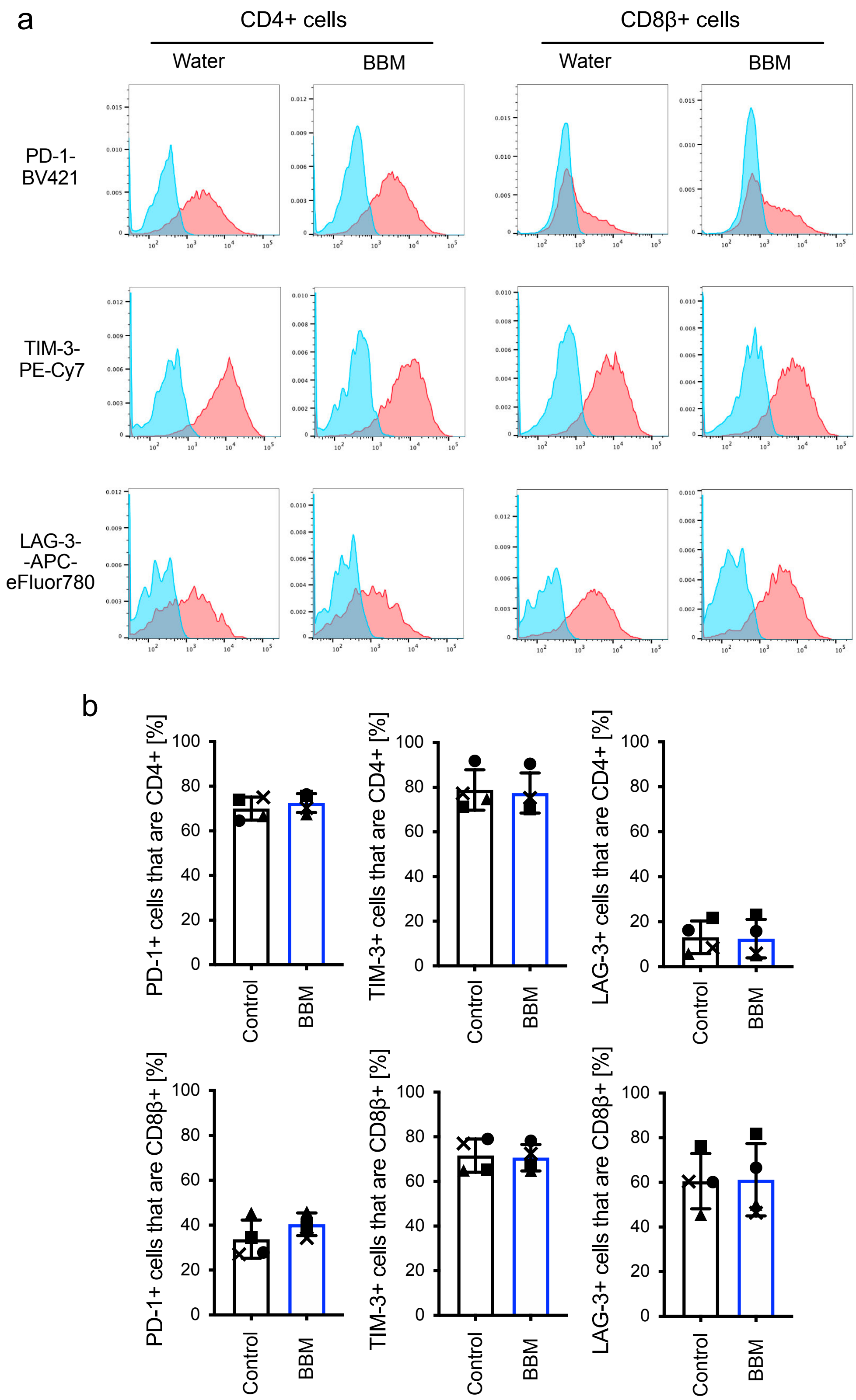

Supplementary Figure 4

(a) Representative FACS plots of T cell exhaustion markers cell cycle phases in CD4+ (left) and CD8β+ (right) cell populations.

(b) Percentages of exhaustion marker-positive cells in CD4+ (top) and CD8β+ (bottom) cell populations. Error bars indicate SD from four donors.

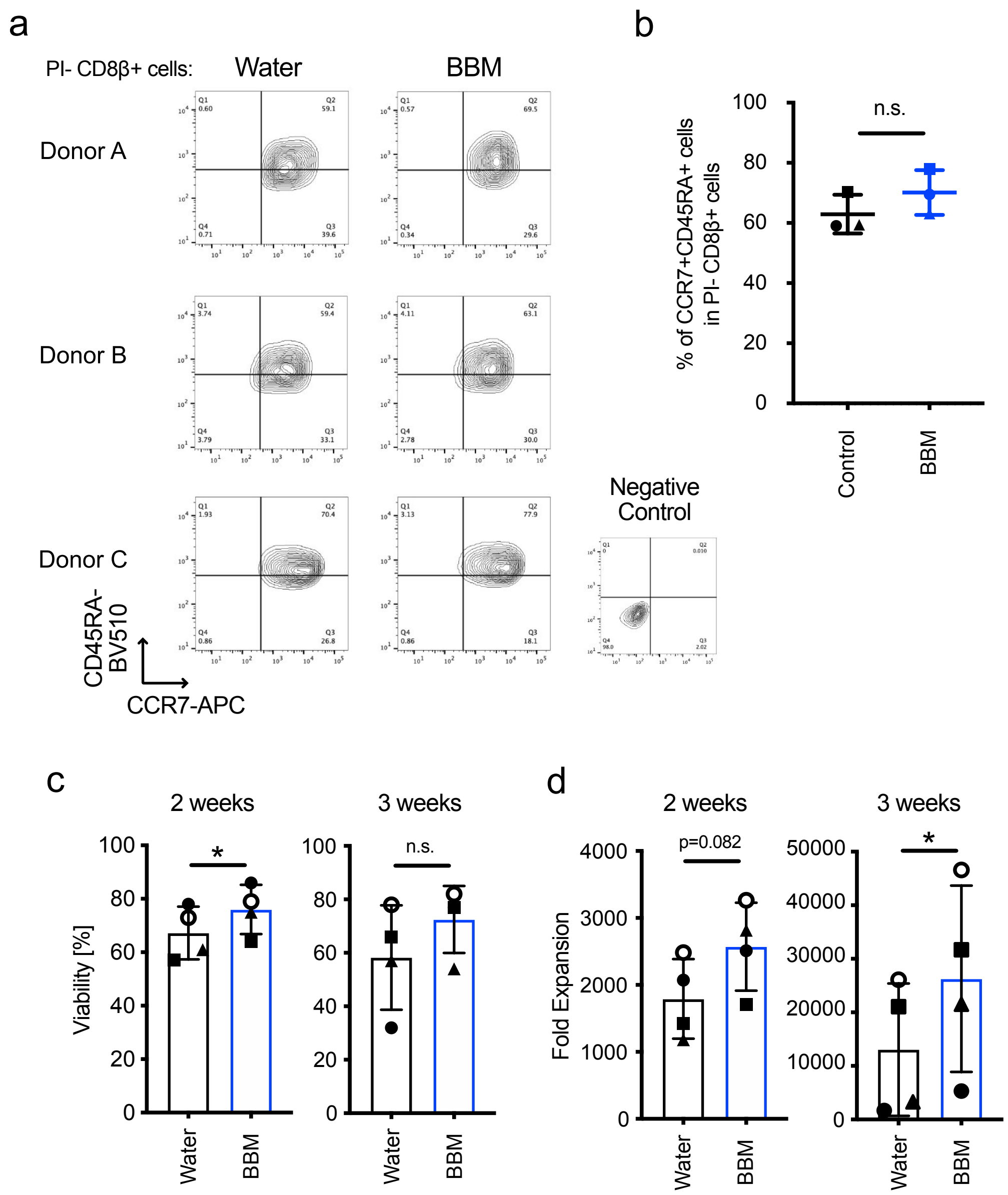

Supplementary Figure 5

(a) Representative FACS plots of the expression of CCR7 and CD45RA at Day 0, the day injected into mouse (see Fig. 3a). PI-CD8β+ cells are shown.

(b) Percentages of CCR7+CD45RA+ cells in CD8β+ fractions at Day 0 (see Fig. 3a).

(c) Viability and fold expansion during long-term culture of primary CD8+ cells at 2 or 3 weeks after CD3/CD28 stimulation. Each data point represents one healthy donor. Error bars indicate SD from three (b) or four (c) donors. Statistical significance is denoted as follows: *, p <0.05; **, p <0.01; ***, p <0.001; and 'n.s.', not significant (paired t-test).

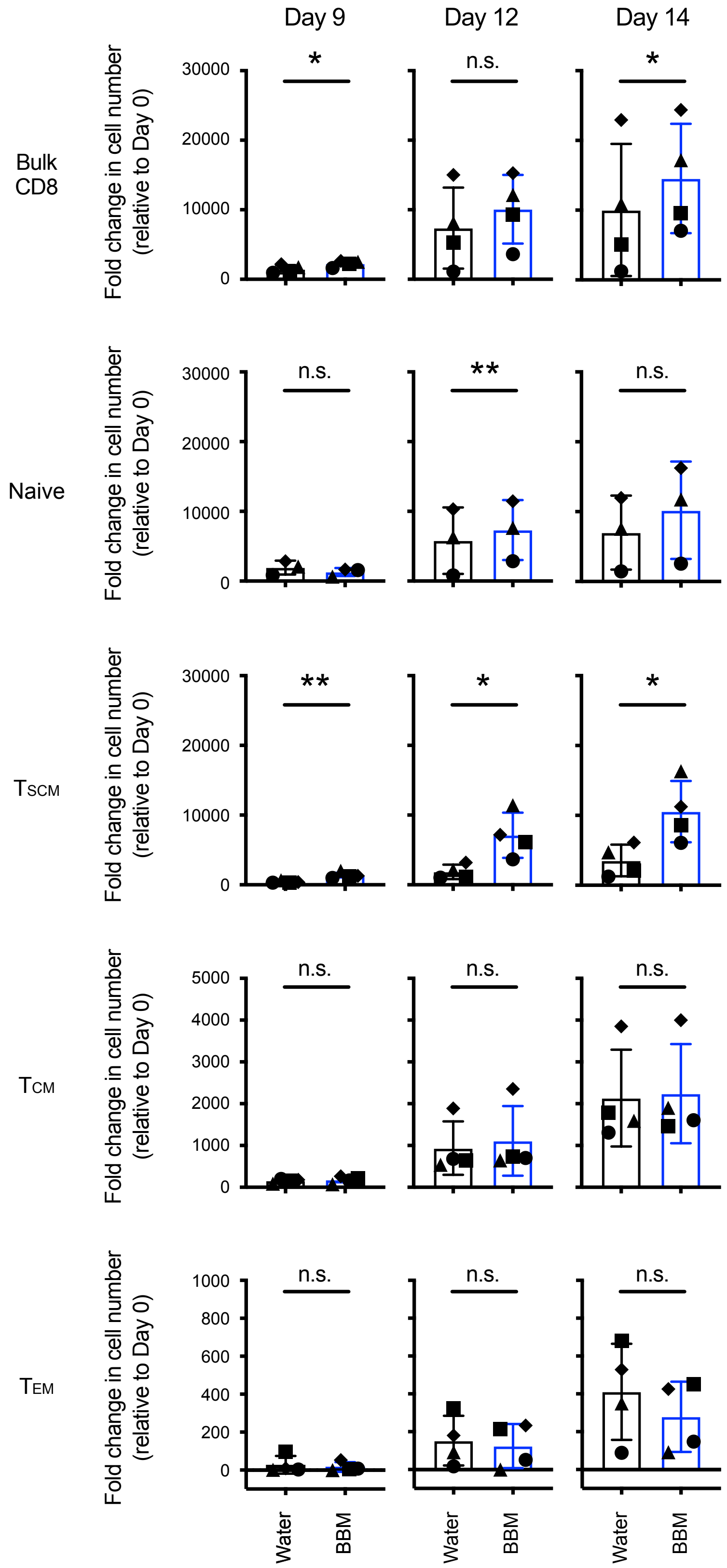

Supplementary Figure 6
Cell proliferation of purified T cell fractions from healthy donor-derived CD8β+ T cells. Bulk CD8β+ cells sorted on FACS was also tested. Error bars indicate SD from three or four donors. Statistical significance is denoted as follows: *, p <0.05; **, p <0.01; ***, p <0.001; and ‘n.s.’, not significant (paired t-test).

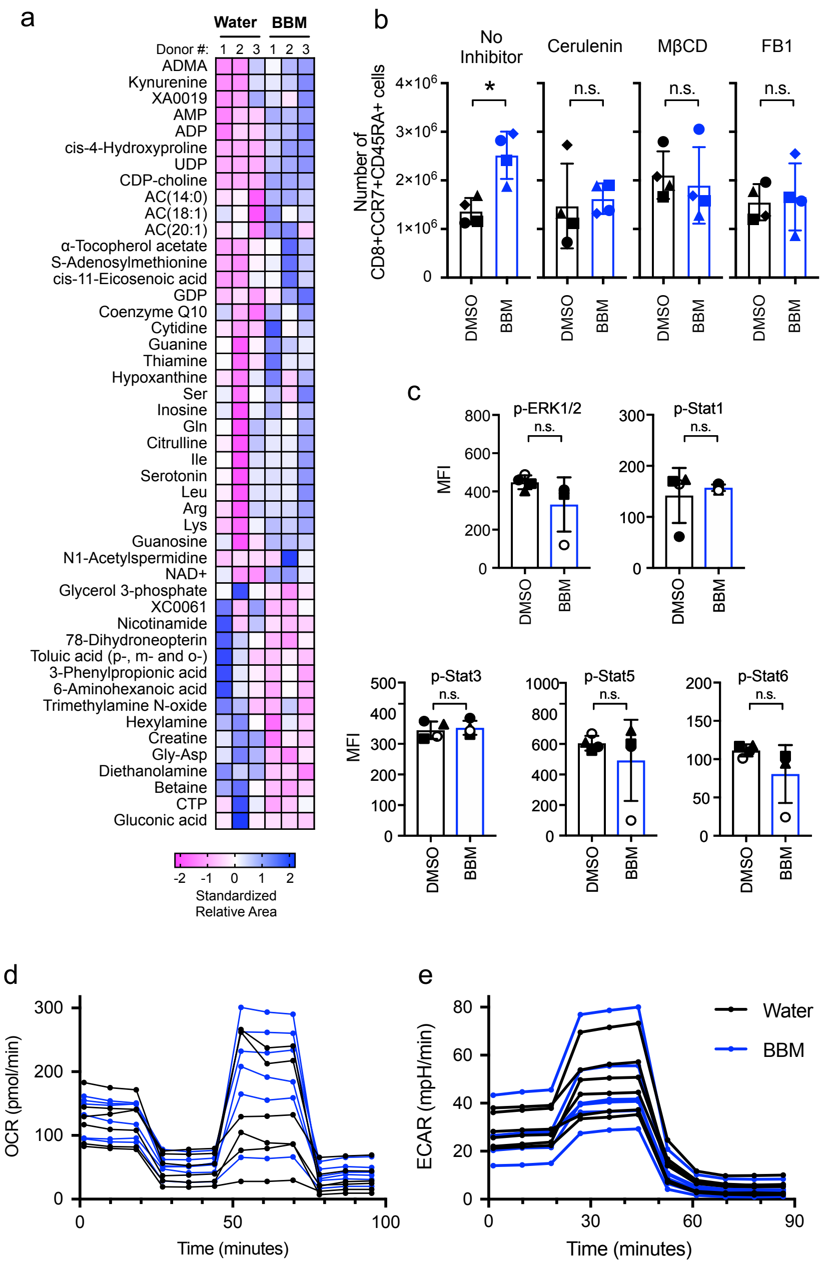

Supplementary Figure 7

(a) Metabolites other than lipids commonly downregulated in all three donors.

(b) Effects of lipid metabolism-related inhibitors in combination with BBM. CD3+ T cells were cultured in the presence of compounds for 14 d.

(c) MFI of phosphorylated proteins in CD8β+ T cells. Error bars indicate SD from four donors.

(d) Oxygen consumption rate (OCR) and (e) extracellular acidification rates (ECAR) across time for BBM-treated CD3+ T cells, related to Figures 5d and 5f, respectively. Each line indicates individual donors.

Statistical significance is denoted as follows: *, $p < 0.05$; **, $p < 0.01$; ***, $p < 0.001$; and ‘n.s.’, not significant (paired t-test). MβCD: Methyl-beta-cyclodextrin, FB1: Fumonisin B1.

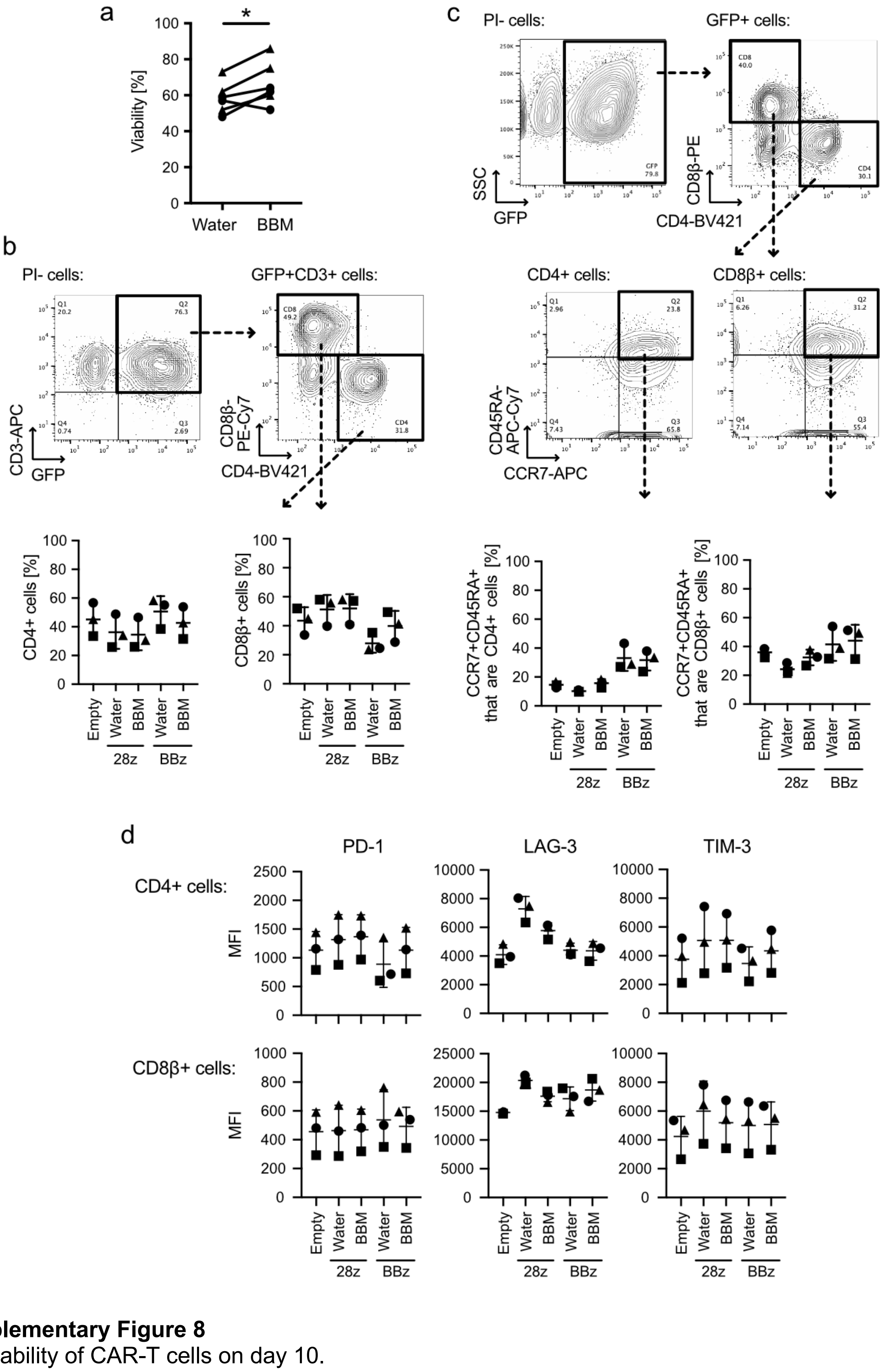

Supplementary Figure 8
(a) Viability of CAR-T cells on day 10.
(b) Percentages of CD4+ and CD8β+ cells in indicated CAR-T cells. (c) Percentages of CCR7+CD45RA+ cells in CD4+ (left) and CD8β+ (right) cell populations in indicated CAR-T cells. (e) MFI of PD-1, LAG-3, or TIM-3 in CD4+ (top) and CD8β+ (bottom) cell populations in CAR-T cells. Error bars indicate SD from three donors. Statistical significance is denoted as follows: *, p <0.05; **, p <0.01; ***, p <0.001; and ‘n.s.’, not significant (paired t-test).

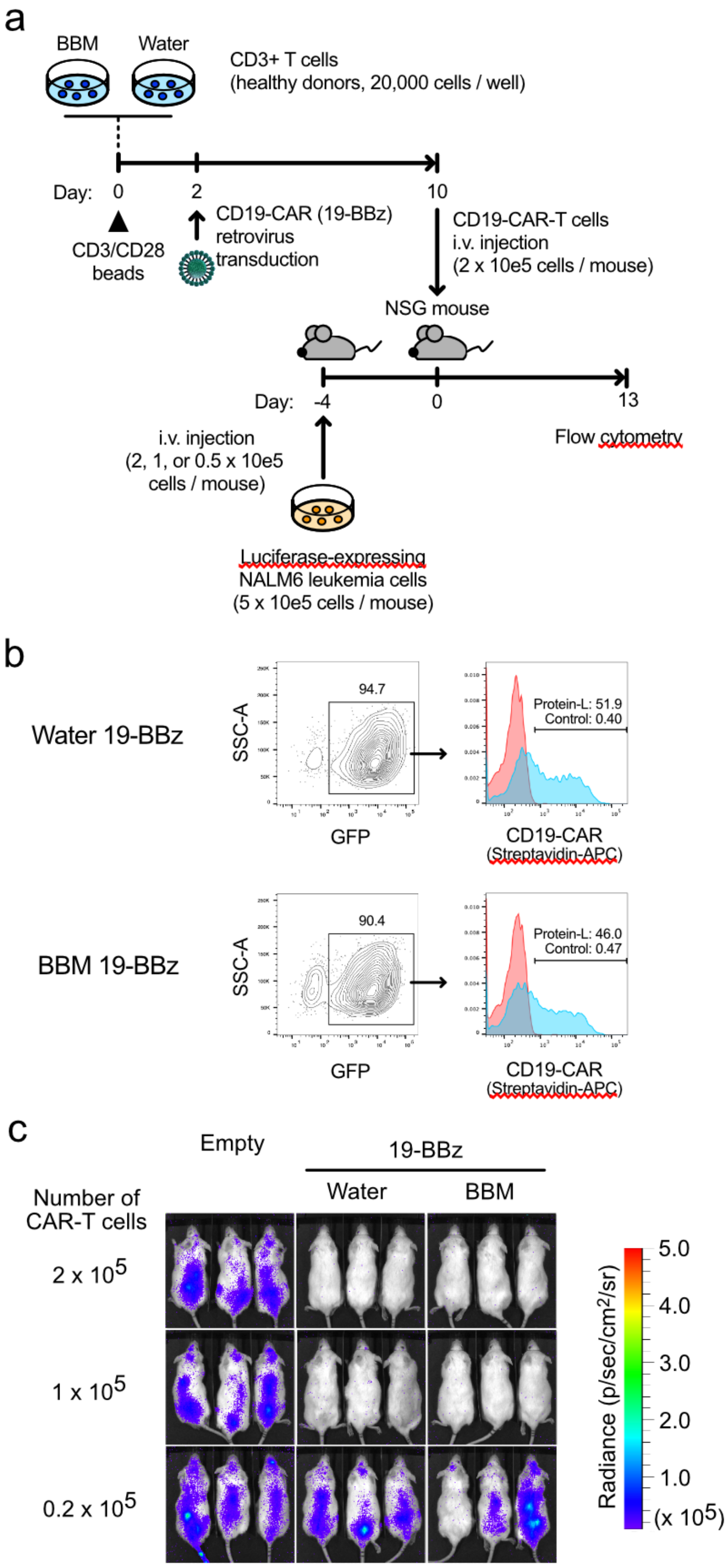

Supplementary Figure 9
(a) Experimental scheme to compare the CAR-T cell numbers to inject into NALM6/NSG mice. (b) Representative flow cytometry plots that depict surface expression of CD19-CAR protein (19-BBz) on day 10. (c) IVIS images of mice that were injected with 19-BBz CAR-T cells thirteen days after the injection.

Supplementary Table 1
The DNA sequences of the anti-CD19 chimeric antigen receptors used in this study.

CAR	DNA sequence
19-28z	ATGGCGCTGCCGGTGACCGCGCTGCTGCTGCCGCTGGCGCTGCTGCTGCATGCGGCGCGCC<br>CGGATATTCAGATGACCCAGACCACCAGCAGCCTGAGCGCGAGCCTGGGCGATCGCGTGACCA<br>TTAGCTGCCGCGCGAGCCAGGATATTAGCAAATATCTGAACTGGTATCAGCAGAAACCGGATGG<br>CACCGTGAAACTGCTGATTTATCATACCAGCCGCCTGCATAGCGGCGTGCCGAGCCGCTTTAGC<br>GGCAGCGGCAGCGGCACCGATTATAGCCTGACCATTAGCAACCTGGAACAGGAAGATATTGCGA<br>CCTATTTTTGCCAGCAGGGCAACACCCTGCCGTATACCTTTGGCGGCGGCACCAAACTGGAAAT<br>TACCGGCGGCGGCGGCAGCGGCGGCGGCGGCAGCGGCGGCGGCGGCAGCGAAGTGAAACT<br>GCAGGAAAGCGGCCCGGGCCTGGTGGCGCCGAGCCAGAGCCTGAGCGTGACCTGCACCGTG<br>AGCGGCGTGAGCCTGCCGGATTATGGCGTGAGCTGGATTCGCCAGCCGCCGCGCAAAGGCCT<br>GGAATGGCTGGGCGTGATTTGGGGCAGCGAAACCACCTATTATAACAGCGCGCTGAAAAGCCG<br>CCTGACCATTATTAAAGATAACAGCAAAAGCCAGGTGTTTCTGAAAATGAACAGCCTGCAGACCG<br>ATGATACCGCGATTTATTATTGCGCGAAACATTATTATTATGGCGGCAGCTATGCGATGGATTATTG<br>GGGCCAGGGCACCAGCGTGACCGTGAGCAGCATTGAAGTGATGTATCCGCCGCCGTATCTGGA<br>TAACGAAAAAAGCAACGGCACCATTATTCATGTGAAAGGCAAACATCTGTGCCCGAGCCCGCTG<br>TTTCCGGGCCCGAGCAAACCGTTTTGGGTGCTGGTGGTGGTGGGCGGCGTGCTGGCGTGCTA<br>TAGCCTGCTGGTGACCGTGGCGTTTATTATTTTTTGGGTGCGCAGCAAACGCAGCCGCCTGCTG<br>CATAGCGATTATATGAACATGACCCCGCGCCGCCCGGGCCCGACCCGCAAACATTATCAGCCGT<br>ATGCGCCGCCGCGCGATTTTGCGGCGTATCGCAGCAGAGTGAAGTTCAGCAGGAGCGCAGAC<br>GCCCCCGCGTACAAGCAGGGCCAGAACCAGCTCTATAACGAGCTCAATCTAGGACGAAGAGAG<br>GAGTACGATGTTTTGGACAAGAGACGTGGCCGGGACCCTGAGATGGGGGGAAAGCCGAGAAG<br>GAAGAACCCTCAGGAAGGCCTGTACAATGAACTGCAGAAAGATAAGATGGCGGAGGCCTACAGT<br>GAGATTGGGATGAAAGGCGAGCGCCGGAGGGGCAAGGGGCACGATGGCCTTTACCAGGGTCT<br>CAGTACAGCCACCAAGGACACCTACGACGCCCTTCACATGCAGGCCCTGCCCCCTCGC
19-BBz	ATGGCCTTACCAGTGACCGCCTTGCTCCTGCCGCTGGCCTTGCTGCTCCACGCCGCCAGGCCG<br>GACATCCAGATGACACAGACTACATCCTCCCTGTCTGCCTCTCTGGGAGACAGAGTCACCATCA<br>GTTGCAGGGCAAGTCAGGACATTAGTAAATATTTAAATTGGTATCAGCAGAAACCAGATGGAACT<br>GTTAAACTCCTGATCTACCATACATCAAGATTACACTCAGGAGTCCCATCAAGGTTCAGTGGCAG<br>TGGGTCTGGAACAGATTATTCTCTCACCATTAGCAACCTGGAGCAAGAAGATATTGCCACTTACTT<br>TTGCCAACAGGGTAATACGCTTCCGTACACGTTCGGAGGGGGGACCAAGCTGGAGATCACAGG<br>TGGCGGTGGCTCGGGCGGTGGTGGGTCGGGTGGCGGCGGATCTGAGGTGAAACTGCAGGAG<br>TCAGGACCTGGCCTGGTGGCGCCCTCACAGAGCCTGTCCGTCACATGCACTGTCTCAGGGGTC<br>TCATTACCCGACTATGGTGTAAGCTGGATTCGCCAGCCTCCACGAAAGGGTCTGGAGTGGCTGG<br>GAGTAATATGGGGTAGTGAAACCACATACTATAATTCAGCTCTCAAATCCAGACTGACCATCATCA<br>AGGACAACTCCAAGAGCCAAGTTTTCTTAAAAATGAACAGTCTGCAAACTGATGACACAGCCATT<br>TACTACTGTGCCAAACATTATTACTACGGTGGTAGCTATGCTATGGACTACTGGGGCCAAGGAAC<br>CTCAGTCACCGTCTCCTCAACCACGACGCCAGCGCCGCGACCACCAACACCGGCGCCCACCA<br>TCGCGTCGCAGCCCCTGTCCCTGCGCCCAGAGGCGTGCCGGCCAGCGGCGGGGGGCGCAGT<br>GCACACGAGGGGGCTGGACTTCGCCTGTGATATCTACATCTGGGCGCCCTTGGCCGGGACTTG<br>TGGGGTCCTTCTCCTGTCACTGGTTATCACCCTTTACTGCAAACGGGGCAGAAAGAAACTCCTG<br>TATATATTCAAACAACCATTTATGAGACCAGTACAAACTACTCAAGAGGAAGATGGCTGTAGCTGC<br>CGATTTCCAGAAGAAGAAGAAGGAGGATGTGAACTGAGAGTGAAGTTCAGCAGGAGCGCAGAC<br>GCCCCCGCGTACAAGCAGGGCCAGAACCAGCTCTATAACGAGCTCAATCTAGGACGAAGAGAG<br>GAGTACGATGTTTTGGACAAGAGACGTGGCCGGGACCCTGAGATGGGGGGAAAGCCGAGAAG<br>GAAGAACCCTCAGGAAGGCCTGTACAATGAACTGCAGAAAGATAAGATGGCGGAGGCCTACAGT<br>GAGATTGGGATGAAAGGCGAGCGCCGGAGGGGCAAGGGGCACGATGGCCTTTACCAGGGTCT<br>CAGTACAGCCACCAAGGACACCTACGACGCCCTTCACATGCAGGCCCTGCCCCCTCGC
