# Supplementary material for: A culture method with berbamine, a plant alkaloid, enhances CAR-T cell efficacy through modulating cellular metabolism
Source: Commun Biol. 2024 Jun 4;7:685. doi: 10.1038/s42003-024-06297-0 (PMC11150386; doi:10.1038/s42003-024-06297-0)
Supplement: Supplementary file 2 — Description of Additional Supplementary Files [file 42003_2024_6297_MOESM2_ESM.pdf]

Description of Additional Supplementary Files

File name: Supplementary Data 1

Description: The source data behind the graphs in the paper

File name: Supplementary Data 2

Description: The analyzed data from RNA-seq (Expected counts, Fig. 4bc)

File name: Supplementary Data 3

File name: The analyzed data from RNA-seq (TPM, Fig. 4d)
